# Photocrosslinkable Chitosan Quaternary Ammonium Salt-Based Ternary Hydrogel with Fibroblast Growth Factor 21 for Diabetic Wound Healing

**DOI:** 10.34133/bmr.0309

**Published:** 2026-01-27

**Authors:** Jingying Hu, Yongqi Xu, Danni Zhou, Kaixuan Chen, Jinwen Jiang, Min Lin, Wenjie Chen, Jing Wu, Hongde Jiang, Mengxiang Zhu, Bin Zhang, Kailei Xu, Peng Wei

**Affiliations:** ^1^ Department of Plastic Surgery, The Affiliated People’s Hospital of Ningbo University, Ningbo 315040, China.; ^2^ Health Science Center, Ningbo University, Ningbo 315211, China.; ^3^ Center for Medical and Engineering Innovation, Central Laboratory, The First Affiliated Hospital of Ningbo University, Ningbo, Zhejiang 315010, China.; ^4^ Department of Hand and Foot Microsurgery, Yuyao People Hospital, Yuyao, Zhejiang 315400, China.

## Abstract

Diabetic wounds represent a critical public health challenge due to impaired healing processes driven by chronic inflammation, infection, and biomechanical deficiencies. Despite advances in wound dressings and negative-pressure therapy, current treatments often fail to provide sufficient mechanical support or to fully resolve inflammatory responses, resulting in prolonged ulceration and high risk of complications. To address these limitations, a photocrosslinkable chitosan quaternary ammonium salt (CQS) derivative (methacrylated CQS [CQS-MA]) was developed to accelerate gelation and improve structural integrity. We then used ultraviolet-initiated copolymerization of CQS-MA with gelatin methacrylate (GelMA) and type I collagen to fabricate a ternary composite hydrogel encapsulating fibroblast growth factor 21 (FGF-21), termed G/C-CS@FGF-21. This composite hydrogel synergistically combined FGF-21’s early-stage inflammation-resolving activity, CQS’s sustained antimicrobial function, GelMA’s tunable mechanical resilience, and collagen’s native cell-adhesive ligands, which could promote all phases of wound repair. In vitro, G/C-CS@FGF-21 promoted macrophage polarization toward the anti-inflammatory M2 phenotype and enhanced fibroblast proliferation and migration. In a full-thickness diabetic mouse wound-healing model, treatment with G/C-CS@FGF-21 accelerated wound closure by mitigating inflammation and promoting reepithelialization and angiogenesis. These findings suggest that the G/C-CS@FGF-21 hydrogel holds strong potential for future clinical translation in diabetic wound management.

## Introduction

Diabetes has emerged as a notable global health challenge in the 21st century, exhibiting one of the fastest-growing trajectories worldwide. From 2000 to 2021, its occurrence rose from 4.6% to 10.5% across the globe, impacting over 537 million individuals [1]. Among the serious complications linked to diabetes, diabetic foot ulcers are particularly severe, affecting an estimated 18.6 million people annually around the world [2]. The management of diabetic foot ulcers is fraught with major difficulties, largely attributable to hyperglycemia. Elevated blood sugar levels intensify persistent inflammatory responses and hinder the shift from inflammation to proliferation in the healing process of wounds. As a result, ulcers that fail to heal properly often develop, exhibiting typical features such as swelling, discomfort, and repeated infections [3]. These circumstances lead to a considerable financial strain on healthcare systems, largely due to extended hospital stays and the necessity for aggressive treatment regimens. Furthermore, they severely impair the overall well-being and daily living standards of affected individuals.

In the clinical management of diabetic wounds, conventional approaches continue to be commonly employed. Standard interventions include surgical debridement, the application of negative-pressure wound therapy, specialized wound dressings, and the utilization of skin grafts [2]. Negative-pressure wound therapy, while effective, requires specialized equipment and trained personnel, thereby increasing the financial burden on patients and healthcare systems [4]. Wound dressings, such as gauze, synthetic fiber cloth, petroleum jelly gauze, and paraffin gauze, are recognized for their cost-effectiveness, practicality, and adaptability. However, they suffer from notable limitations, such as poor exudate absorption, frequent need for replacement, excessive permeability that can lead to wound dehydration, adhesion to the wound bed that risks mechanical trauma upon removal, and an inability to maintain a consistently moist healing environment [5]. Skin grafting, a cornerstone in reconstructive surgery, involves transferring healthy skin from a donor site to cover damaged areas. Despite its therapeutic value, the procedure carries inherent risks, including donor-site morbidity, size mismatch, and sensory dysfunction.

In the field of wound care, biomaterials are attracting increasing attention owing to their beneficial properties [6–9]. These advantages, such as the ability to maintain a moist environment, high biocompatibility, and capacity for biodegradation, have prompted growing interest in their therapeutic application in recent years [10–12]. Chitosan, a naturally derived polymer, demonstrates inherent antibacterial and anti-inflammatory characteristics, alongside favorable biocompatibility and biodegradability. These properties have led to its extensive application within the field of tissue engineering.

However, chitosan has poor water solubility and low bioactivity, necessitating chemical modifications to broaden its range of applications [13]. Among its derivatives, chitosan quaternary ammonium salt (CQS), a chemically modified derivative of chitosan in which quaternary ammonium groups are introduced onto the chitosan backbone, exhibits markedly improved hydrophilicity and enhanced antimicrobial efficacy [14]. Despite these advantages, CQS suffers from suboptimal gelation, excessive swelling, and diminished cell-adhesion capacity, which constrain its utility in advanced wound-healing constructs. Gelatin methacrylate (GelMA), a photocrosslinkable hydrogel derived from gelatin, offers a fast gelation rate, mechanical strength, and cellular adhesion properties [15]. Nevertheless, the process of denaturation causes gelatin and its derivatives to forfeit collagen’s distinctive triple-helix conformation, a structure essential for initiating cellular signaling pathways and supporting key biological interactions within the extracellular matrix (ECM) [16].

Taking advantage of their high water content, hydrogels can easily encapsulate hydrophilic drugs and achieve controlled release during the wound-healing process. Fibroblast growth factor 21 (FGF-21), an adipokine belonging to a specific FGF subfamily, is acknowledged as a potent metabolic regulator of glucose and lipid homeostasis [17]. Additionally, it has been demonstrated to modulate macrophage migration, control inflammatory processes, and influence lipid metabolism [18]. Moreover, FGF-21 demonstrates therapeutic promise for diabetic wound repair by stimulating angiogenesis in endothelial cells via mechanisms involving PPARγ activation [19]. It further facilitates epidermal cell migration and differentiation through autophagy processes regulated by SIRT1 [20].

**Fig. 1.**
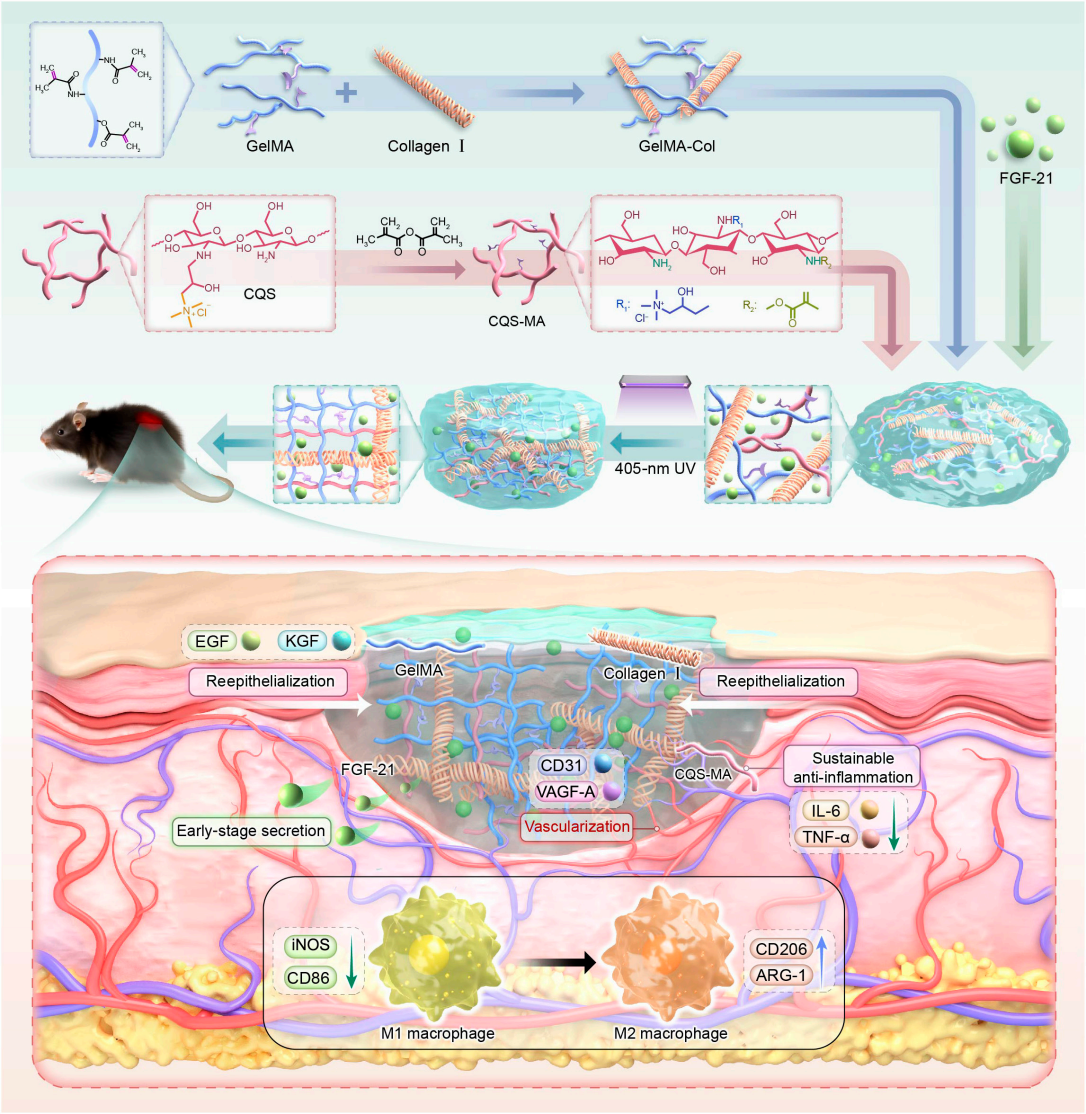
Schematic diagram of the development of G/C-CS@FGF-21 hydrogel for diabatic wound healing. GelMA, gelatin methacrylate; Col, type I collagen; CQS, chitosan quaternary ammonium salt; CQS-MA, methacrylated CQS; FGF-21, fibroblast growth factor 21; UV, ultraviolet; EGF, epidermal growth factor; KGF, keratinocyte growth factor; VEGF-A, vascular endothelial growth factor A; IL-6, interleukin-6; TNF-α, tumor necrosis factor-α; iNOS, inducible nitric oxide synthase; ARG-1, arginase 1.

For the present investigation, a hydrogel was fabricated through the methacrylation of CQS (yielding methacrylated CQS [CQS-MA]), thereby conferring photocrosslinking functionality while improving both the gel formation kinetics and the mechanical integrity of the resulting network (Fig. 1). We subsequently fabricated a ternary composite wound-healing hydrogel by copolymerizing CQS-MA with GelMA and native type I collagen under ultraviolet irradiation, thereby synergistically integrating the long-term antimicrobial properties of CQS, tunable mechanical resilience of GelMA, and intrinsic cell-adhesive ligands of collagen. To further endow the scaffold with pro-regenerative functionality, FGF-21 was encapsulated within the interpenetrating network for early-stage inflammation resolution and accelerated dermal tissue regeneration.

## Materials and Methods

### Materials

High-purity CQS (S26618) was supplied by Yuanye Bio-Technology (Shanghai, China). Gelatin (G108395) and methacrylic anhydride (M102519) were purchased from Aladdin Industrial Corporation (Shanghai, China). Type I collagen (Catalog No. 5005, 100 ml, 3 mg/ml) was obtained from Advanced Biomatrix (Carlsbad, CA, USA). Recombinant human FGF-21 (HY-P717) was sourced from MedChemExpress (Monmouth Junction, NJ, USA). The photoinitiator lithium phenyl-2,4,6-trimethylbenzoylphosphinate (LAP, EFL-LAP) was procured from EngineeringForLife (Suzhou, China). The mouse fibroblast cell line L929 and the murine macrophage cell line RAW264.7 were acquired from Procell Life Sciences (Shanghai, China) and Cas9X Biotech (Suzhou, China), respectively. All cell lines were cultured under standard conditions and consistently tested negative for mycoplasma contamination using MycoBlue Mycoplasma Detector (D101-01, Vazyme, Nanjing, China). Dulbecco’s modified Eagle medium (DMEM, high glucose, with pyruvate, C11995500BT), penicillin–streptomycin (10,000 U/ml, 15140122), and 0.25% trypsin–EDTA (25200056) were supplied by Thermo Fisher Scientific (Waltham, MA, USA). A specialized medium for RAW264.7 cells (TCM-G766) was also utilized. Additional reagents included phosphate-buffered saline (PBS; P1020), 4′,6-diamidino-2-phenylindole (DAPI; C0060), 4% paraformaldehyde (PFA; P1110), Live/Dead Cell Staining Kit (CA1630), and phalloidin solution (CA1640) from Solarbio Science & Technology (Beijing, China). Cell Counting Kit-8 (CCK-8; C0038) was obtained from Beyotime Biotechnology (Shanghai, China). RNA extraction was performed using TRIzol reagent (15596018, Life Technologies, MA, USA). Reverse transcription was carried out with the FastKing gDNA Dispelling RT SuperMix kit (KR118, Tiangen, Beijing, China), and quantitative real-time polymerase chain reaction (qRT-PCR) was conducted using Power SYBR Green PCR Master Mix (4367659, Life Technologies). The protein concentration was determined with the BCA Protein Assay Kit (Thermo Scientific, MA, USA). Fetal bovine serum was acquired from ExCell Bio (Shanghai, China). All oligonucleotide primers were synthesized by Shanghai Sangong Biotechnology Co., Ltd. The following primary antibodies were used: platelet-derived growth factor subunit A (PDGFA; AF5179), platelet-derived growth factor subunit B (PDGFB; AF0240), interleukin-6 (IL-6; DF6087), alpha smooth muscle actin (α-SMA; AF1032), keratinocyte growth factor (KGF; DF13342), and tumor necrosis factor-α (TNF-α; AF7014) from Affinity Biosciences (Jiangsu, China); vascular endothelial growth factor A (VEGF-A; AB214424) from Abcam (Cambridge, UK); CD31 (A0378), tubulin (AC012), β-actin (AC004), and epidermal growth factor (EGF; A13615) from ABclonal (Wuhan, China); and integrin beta 1 (12594-1-AP), CD86 (13395-1-AP), inducible nitric oxide synthase (iNOS; 18985-1-AP), CD206 (18704-1-AP), and arginase 1 (ARG-1; 16001-1-AP) from Proteintech Group (Wuhan, China). Secondary antibodies included Goat Anti-Rabbit IgG H&L (ab150077) and Goat Anti-Mouse IgG H&L (ab150113) from Abcam (Cambridge, UK), as well as a horseradish peroxidase (HRP)-conjugated secondary antibody (BL001A) from Biosharp Life Sciences (Anhui, China).

### Synthesis of GelMA

A 20 wt% gelatin solution was prepared in a buffer (174.4 mM Na_2_CO_3_ and 75.0 mM NaHCO_3_) with continuous stirring at 50 °C for 2 h. To this solution, 0.3% (v/v) methacrylic anhydride was introduced, and the reaction was protected from light while stirring at 50 °C for another 2 h. The resulting mixture was diluted 10-fold using ultrapure water and then dialyzed for a 3-d period at ambient temperature. Throughout this process, the dialysis buffer was refreshed 3 times daily. Following purification, the product was lyophilized and stored at −20 °C until future application.

The methacrylation substitution degree in the synthesized GelMA was measured via a TNBS (2,4,6-trinitrobenzenesulfonic acid) assay following an established protocol [21]. In brief, both GelMA and unmodified gelatin (*n* = 3) were solubilized at 1.6 mg/ml in 0.1 M sodium bicarbonate buffer. Subsequently, 500 μl of each protein solution was combined with an equal volume of 0.1% TNBS reagent and subjected to incubation at 37 °C for 2 h. The reaction was stopped using 250 μl of 1 M HCl and 500 μl of 10 wt% sodium dodecyl sulfate (SDS). Absorbance measurements were taken at 335 nm. The free amine group concentration was subsequently determined using a pre-generated glycine standard curve covering concentrations from 0 to 100 μg/ml. The methacrylation degree of GelMA was determined to be approximately 30% using this approach.

### Synthesis of methacrylate anhydride-grafted quaternary ammonium chitosan

To synthesize CQS-MA, CQS (4 wt%) was dissolved in PBS by stirring at room temperature overnight. To synthesize CQS-MA, methacrylic acid (2% v/v) was introduced into the quaternary ammonium chitosan solution, followed by stirring under dark conditions at ambient temperature for 1.5 h. Then, the reacted solution was diluted 10-fold using ultrapure water and dialyzed at ambient temperature for a 3-d period, replacing the dialysis water 3 times per day. Finally, the dialyzed product was lyophilized and stored at −20 °C until future application.

### Preparation of composite hydrogels

GelMA at concentrations of 5, 10, and 15 wt% was mixed into a complete culture medium supplemented with 0.5 wt% LAP under 37 °C conditions (group G). On ice, collagen was mixed with 10× DMEM (8:1 v:v) and neutralized with sterile 1 M NaOH. This collagen solution was combined with GelMA at room temperature to yield precursors with 5 wt% GelMA and either 0.12 wt% collagen (G/C1) or 0.16 wt% collagen (G/C2). Separately, CQS-MA was dissolved in the same medium with 0.5 wt% LAP at room temperature and added to G/C2 to a final concentration of 1 wt% G/C-CS). FGF-21 (100 ng/ml) was then incorporated into this mixture (G/C-CS@FGF-21). Each precursor was cast into 8 mm × 1 mm glass molds and crosslinked under 405-nm ultraviolet light to form disk-shaped hydrogels.

### Rheology and mechanical properties

Rheological characterization of the pre-gel solutions (*n* = 3 per formulation) was performed using HAAKE MARS iQ Air Rheometer (Thermo Scientific) equipped with a 25-mm-diameter parallel plate geometry. Measurements of viscosity were conducted across a thermal profile ranging from 4 to 35 °C, as well as under varying shear rate conditions. The storage (*G*′) and loss (*G*″) moduli were measured at a fixed shear rate and a constant frequency.

### Hemolysis assay

Fresh rabbit blood and physiological saline were combined in equal volumes (1:1, v/v). The resulting mixture was then centrifuged at 1,000 rpm for a 10-min duration. The collected red blood cells (RBCs) underwent 3 rounds of washing with saline and were ultimately diluted to a 5% (v/v) suspension. Each hydrogel precursor (500 μl) was combined in a 1:1 ratio with the RBC suspension and transferred into a 24-well plate (*n* = 3 per group). The samples were gently shaken at 100 rpm and subjected to incubation at 37 °C for 60 min. Negative control groups received saline instead of hydrogel, while positive controls were treated with 0.1% Triton X-100. Following incubation, the supernatant was collected for another centrifugation step (1,000 rpm, 10 min); 100 μl of the clarified supernatant from each sample was transferred to a 96-well plate. The absorbance at 540 nm was measured using a spectrophotometer. The percentage of hemolysis was determined using the following equation: Hemolysis (%) = (ODs − ODn)/(ODp − ODn) × 100%.

Here, ODs denotes the absorbance of the supernatant after the hydrogel was incubated with the RBC suspension, ODn refers to the absorbance from the physiological saline control group, and ODp corresponds to the absorbance of the Triton X-100-treated positive control.

### Scanning electron microscopy

After photocrosslinking, all hydrogel formulations (*n* = 3 per group) were freeze-dried and sputter-coated with a thin gold layer (ISC 150, Supro Instrument, NY, USA) to improve imaging quality. Surface morphology was assessed by scanning electron microscopy (Phenom Pro, the Netherlands), and quantitative analysis of pore size was performed using ImageJ.

### Compression testing

Compression assays were performed on hydrogel samples (*n* = 3 per group) at room temperature with an AGS-X universal mechanical testing system (Shimadzu, Japan). The hydrogels were fabricated into cylindrical shapes measuring 2 mm in thickness and 8 mm in diameter. The elastic modulus under compression was determined from the gradient of the linear elastic region (0% to 30% strain) in the stress-versus-strain curve.

### Hydrogel stability test

To simulate physiological culture environments, photocrosslinked hydrogel samples (*n* = 3 per group) were placed in complete medium and maintained in a 5% CO_2_ incubator at 37 °C conditions for a period of 7 d. The diameter of individual hydrogels was determined daily utilizing an electronic digital caliper (Deli, Ningbo, China), and values were normalized against their initial diameters to evaluate the swelling ratio.

To evaluate biodegradation, the hydrogels (*n* = 3 per group at each time point) were weighed (*W*_0_), then immersed in 0.25% trypsin–EDTA, and incubated at 37 °C. At 10, 20, 30, 40, 50, 60, 70, and 80 min, the samples were removed from trypsin, gently blotted to eliminate excess surface water, and weighed to obtain *W_t_*. The biodegradation rate was calculated using the following equation:

$$\text{Degradation rate}\;\left(\%\right)=\frac{W_{0}-W_{t}}{W_{0}}\times 100$$ (1)

### Fourier transform infrared spectroscopy

Lyophilized samples of all formulations were analyzed by Fourier transform infrared (FTIR) spectroscopy, with CQS, CQS-MA, and GelMA serving as controls. Infrared spectra were acquired using a Nicolet iS5 FTIR spectrometer (Thermo Nicolet, Waltham, MA) fitted with an iD7 attenuated total reflectance attachment. Measurements were performed at a resolution of 4 cm^−1^ with 16 accumulated scans. A background spectrum was acquired under identical conditions prior to each sample measurement and subtracted from the corresponding sample spectrum.

### Biocompatibility analysis

L929 fibroblast cells were maintained in DMEM supplemented with 10% fetal bovine serum and 1% penicillin–streptomycin. Concurrently, RAW264.7 cells were propagated in a proprietary growth medium formulated for that specific cell type. All cultures were incubated at 37 °C in a humidified atmosphere containing 5% CO_2_. Hydrogels with varying compositions were shaped into disklike forms, positioned in 48-well plates, and then used for cell seeding. A total culture period of 7 d was employed, with the medium being replaced every 2 d. Cell viability was assessed on day 5 using a fluorescent live/dead assay (*n* = 3 per group) based on the manufacturer’s protocol. The hydrogels were washed 3 times using assay buffer, treated with 500 μl of working solution at 37 °C for 15 min, and then rinsed again with the same buffer. Subsequent imaging was carried out with a fluorescence microscope (Leica Microsystems, Germany). Cell proliferation on the hydrogel surfaces was assessed using a CCK-8 assay at time intervals of 1, 3, 5, and 7 d. Following the manufacturer’s protocol, samples were treated with 10% (v/v) CCK-8 working solution and incubated under standard cell culture conditions (37 °C, 5% CO_2_). Subsequently, 100 μl of supernatant from each well was collected and pipetted into a 96-well plate, and the optical density was measured at 450 nm with a microplate reader. Proliferation rates were calculated by normalization against the optical density values measured on day 0 for each sample.

### RNA

Total RNA was extracted using TRIzol reagent (Life, Waltham, MA, USA) following the supplier’s recommended procedures. In brief, chloroform was applied to the samples to induce phase separation. The RNA was then precipitated by adding isopropanol, following 2 washing steps with 75% ethanol; the resulting pellet was finally resuspended in RNase-free water. To quantify the RNA concentration, a NanoDrop spectrophotometer (Thermo Scientific, Waltham, MA, USA) was employed.

### RNA sequencing and bioinformatics analysis

To assess RNA integrity in RAW264.7 cells, Agilent 2100 Bioanalyzer (Agilent Technologies, CA, USA) was employed. The construction of RNA sequencing (RNA-seq) libraries utilized the VAHTS Universal V6 RNA-seq Library Prep Kit, adhering strictly to the supplier’s instructions. For each sample, transcriptome sequencing and initial data processing were performed on the Illumina NovaSeq 6000 system by OE Biotech Co., Ltd. (Shanghai, China), which yielded approximately 50 million 150-bp paired-end reads. Using the fastp tool, raw FASTQ files underwent quality control processing to produce clean reads. These high-quality reads were subsequently aligned to the reference genome using HISAT2. To quantify gene expression, HTSeq-count provided raw read counts, and fragments per kilobase of transcript per million mapped fragments (FPKM) values were used. Reproducibility among samples was evaluated through principal component analysis using R (v3.2.0). To detect significantly differentially expressed genes (DEGs), DESeq2 was used to identify differentially DEGs with a threshold of |log_2_ (fold change)| ≥ 1 and an adjusted *P* value (*Q*) <0.05. Expression profiles across samples were visualized by hierarchical clustering. For functional interpretation, Gene Ontology enrichment analysis of the DEGs was performed using the hypergeometric test, with results depicted as column, chord, and bubble charts in R. Additionally, gene set enrichment analysis was performed using a predefined gene set. Genes were ranked based on their differential expression levels (e.g., log_2_ fold change), and enrichment for the gene set was assessed at the top or bottom of this ranked list.

### Real-time PCR

Complementary DNA was synthesized from total RNA using the FastKing DNA Dispelling RT SuperMix kit (Tiangen Biotech, Beijing, China) according to the manufacturer’s protocol. qRT-PCR amplification was conducted on a Roche instrument (Basel, Switzerland) using 10-μl reactions that comprised Power SYBR Green PCR Master Mix, 20 ng of complementary DNA, and primers specific to target genes, with 3 replicates per primer set.

### Western blotting

Cells grown on hydrogels (L929 and RAW264.7) were detached using trypsin, lysed in radioimmunoprecipitation assay (RIPA) buffer, and centrifuged. The protein levels in the resulting supernatants were quantified via the bicinchoninic acid (BCA) assay. For animal tissue samples, RIPA buffer was added before mechanical disruption with a Tissuelyser-32L homogenizer (Shanghai Jingxin, China), followed by centrifugation, and then, the protein concentration was determined via the BCA assay. Equal amounts of protein were mixed with SDS–polyacrylamide gel electrophoresis (SDS-PAGE) loading buffer and heat-denatured at 100 °C for 10 min. After resolution by 10% SDS-PAGE, proteins were blotted onto polyvinylidene fluoride membranes. Following a 3-h incubation at room temperature in 5% nonfat milk to block nonspecific binding sites, the membranes were incubated with specific primary antibodies overnight at 4 °C. For L929 cells, antibodies included CD31 (1:2,000), EGF (1:1,000), VEGF-A (1:2,000), KGF (1:500), PDGFA (1:2,000), α-SMA (1:1,000), β-actin (1:5,000), and tubulin (1:5,000). For RAW264.7 cells, antibodies against CD86 (1:1,000), iNOS (1:500), IL-6 (1:500), ARG-1 (1:5,000), CD206 (1:500), and TNF-α (1:2,000) were used.

Following washing steps, the membranes were incubated with HRP-conjugated secondary antibodies (diluted 1:5,000) for 1 h at room temperature. Chemiluminescent detection was performed using the Clarity Western ECL Substrate (Bio-Rad, CA, USA), following the manufacturer’s instructions. The signal intensity was analyzed through densitometry and normalized to internal reference proteins.

### Immunofluorescence staining

To evaluate cell morphology on hydrogel surfaces, phalloidin staining was performed. Adhering cells on hydrogels collected on days 1, 3, 5, and 7 were first fixed using 4% PFA, according to the manufacturer’s instructions. Following 3 PBS washes, the samples were treated with 0.5% Triton X-100 for permeabilization (15 min). After that, they were incubated with phalloidin at a dilution of 1:500 for 1 h and nuclei were labeled with DAPI during a 10-min incubation in darkness. Imaging was promptly conducted using a confocal microscope (Nexcope, Ningbo, China).

To perform immunofluorescence staining on in vitro cell cultures, samples were initially subjected to fixation with a 4% (w/v) PFA solution for 15 min. After 3 washes with PBS, permeability was achieved by treating the samples with 0.5% Triton X-100 for 15 min, and then the samples were blocked by incubation in 5% bovine serum albumin (BSA) for 1 h at ambient temperature. For immunolabeling, a solution of primary antibodies in 5% BSA was prepared and applied, including integrin beta 1 (1:200), EGF (1:50), KGF (1:100), PDGFB (1:200), α-SMA (1:200), CD86 (1:300), iNOS (1:500), ARG-1 (1:500), and CD206 (1:500). Overnight incubation was carried out at 4 °C. On the next day, after 3 PBS washes, samples were incubated with appropriately diluted secondary antibodies (1:200) for 1 h at ambient temperature under light-protected conditions. After additional PBS washes, nuclei were labeled with DAPI, followed by 3 final washes. Image acquisition was conducted immediately employing a confocal microscope.

For histological analysis, wound-adjacent skin samples were processed by fixation in 4% PFA, followed by paraffin embedding and sectioning at 8 μm. These slices were placed on glass slides, baked at 60 °C for 50 min to enhance adhesion, and then deparaffinized with xylene and rehydrated through a descending ethanol series into Tris–EDTA buffer. After incubation with primary antibodies targeting TNF-α (1:200) and α-SMA (1:200) at 4 °C overnight, the sections were blocked with 10% BSA for 30 min at room temperature. After thorough washing with PBS, the sections were exposed to secondary antibodies at a dilution of 1:200 for 1 h at room temperature followed by a 10-min incubation with DAPI; the samples were immediately imaged using a laser scanning confocal microscope.

### Cell scratch assay

L929 cells (*n* = 3 per group) grown on various hydrogel formulations were harvested using 0.25% trypsin–EDTA and collected by centrifugation (1,000 rpm, 3 min), reconstituted in medium, and then transferred into 24-well plates. After attaining 90% to 100% confluency, a straight scratch wound was introduced into the monolayer in each well with a sterile pipette tip. Images of the wounded regions were recorded at 0, 24, and 48-h time points utilizing an inverted microscope (Leica Microsystems, Germany). The percentage of wound closure was quantified by comparing the cell-free area to the total area of the field of view.

### Animal experiments and wound closure measurement

The Institutional Animal Care and Use Committee at Ningbo University examined and authorized all research procedures involving animal subjects under approval protocol 12703. A total of 72 C57BL/6 mice (6 to 8 weeks old, sex-balanced) received daily intraperitoneal administrations of streptozotocin at a dosage of 40 mg/kg over 7 d to establish a diabetic model. Hyperglycemia was confirmed when blood glucose levels exceeded 16.8 mmol/l. With an 8-mm biopsy punch, full-thickness excisional wounds were created on the dorsal skin. To restrict wound contraction, a silicone ring with an inner diameter of 1 cm was secured around each wound. The mice were assigned to 4 experimental groups randomly, each containing 18 animals with equal numbers of males and females: blank, G/C, G/C-CS, and G/C-CS@FGF-21. Hydrogels corresponding to each group were applied to the wounds, which were then covered with a vacuum sealing drainage (VSD) membrane to secure the dressing. The control group received sterile physiological saline and was similarly covered with a VSD membrane.

The progression of wound closure was tracked at postoperative days 0, 3, 6, 9, 12, and 15. Wound dimensions were measured with ImageJ, and the percentage of healing was calculated according to the following equation: Healing rate (%) = (*A_n_*/*A*_0_) × 100%, where *A*_0_ corresponds to the initial wound area on day 0 and *A_n_* refers to the area measured at each designated time point thereafter.

### Hematoxylin and eosin, Masson, and Sirius Red staining

Tissue sections were first incubated in hematoxylin solution for a duration of 10 min, for hematoxylin and eosin (H&E) staining and then treated with acid alcohol for differentiation over 3 min, rinsed in deionized water, and counterstained with eosin Y for 1 min. After staining, samples were dehydrated through a graded ethanol series, cleared in xylene, and then finally mounted using resin. Images were acquired with an inverted microscope.

In Masson’s trichrome staining, sections were first incubated with Weigert’s iron hematoxylin solution for 5 min and differentiated in acidic ethanol for 10 s; before being rinsed in distilled water, sections were treated with a bluing agent for 3 min. Following a 5-min incubation in Ponceau–acid fuchsin and a subsequent rinse, the sections were differentiated in phosphomolybdic acid for 1 min prior to a final 1-min counterstain with aniline blue. A 1-min treatment with acetic acid working solution followed. After ethanol dehydration and xylene clearing, samples were sealed with resin. Images were acquired using an inverted microscope and analyzed with the ImageJ software to determine muscle fiber cross-sectional area.

For Sirius Red staining, sections were incubated in a saturated 0.1% (w/v) Sirius Red solution at ambient temperature for 60 min. They were washed twice to remove unbound dye with 0.5% acetic acid. For permanent preservation, sections were dehydrated through an ascending ethanol gradient, cleared in 2 changes of xylene to ensure complete removal of alcohol, and finally coverslipped with a synthetic mounting medium. Imaging was performed using an inverted microscope, and the collagen content was quantified with the ImageJ software.

### Data analysis

Unless explicitly indicated, all data were analyzed using GraphPad Prism 8.02. Experiments were conducted with 3 biologically independent replicates, and 9 samples in total were included for averaging. Values are reported as mean ± standard deviation. Using the Student *t* test, group comparisons were made and statistical significance was set at a threshold of *P* < 0.05. Additionally, GraphPad Prism (v8.02) was utilized for all statistical analyses. Statistical significance was determined using the following criteria: all data are expressed as mean ± standard deviation (SD) (*n* = 3). *P < 0.05, **P < 0.01, and ***P < 0.001 indicate significant differences compared with group G; ns denotes no significant difference.

## Results and Discussion

### Material characterization of composite hydrogels

**Fig. 2.**
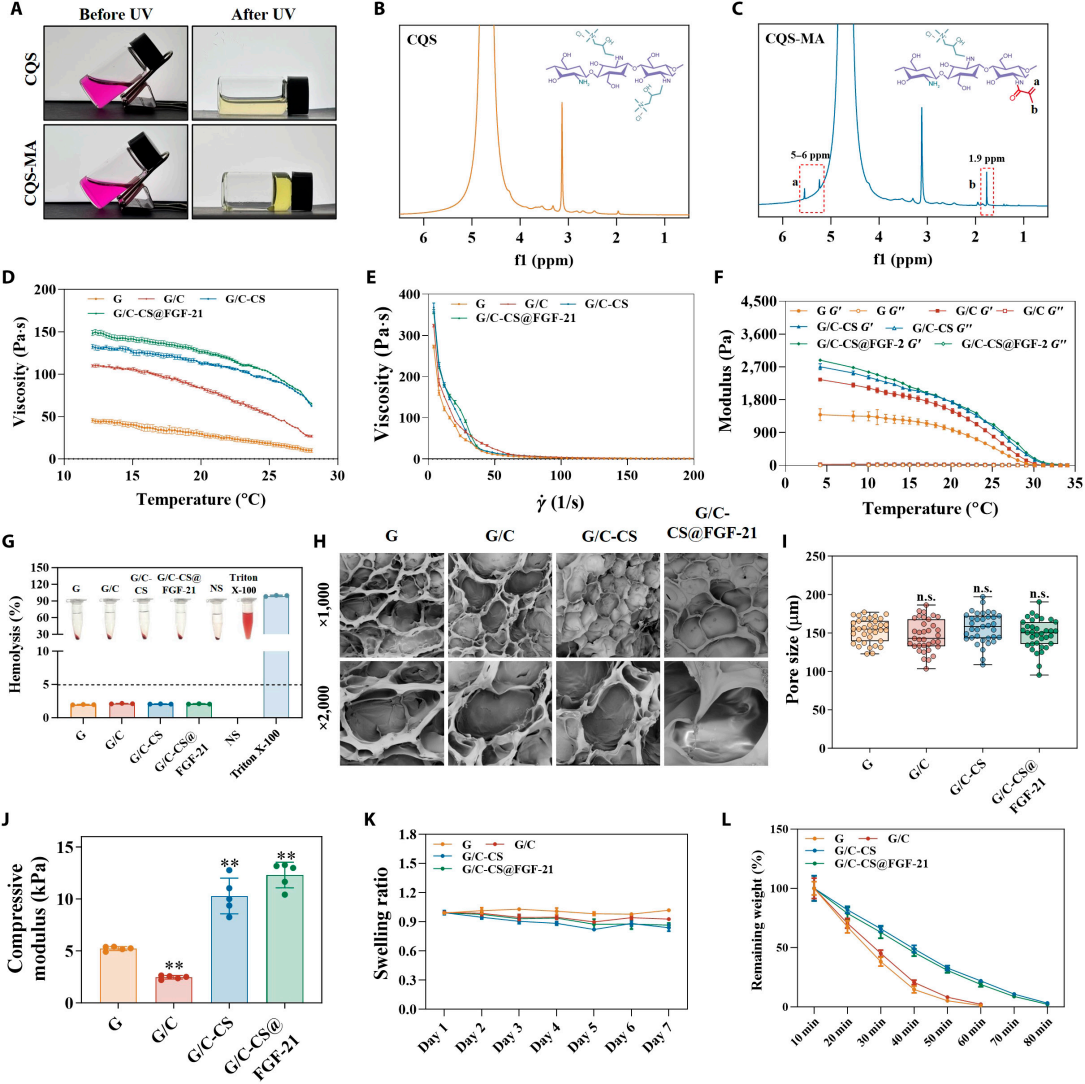
Material characterization. (A) Photos of CQS and CQS-MA after photocrosslinking. (B and C) Nuclear magnetic resonance (NMR) of CQS and CQS-MA. (D) Temperature-dependent viscosity profiles of precursor solutions. (E) Shear-rate-responsive viscosity behavior of precursors. (F) Thermal variations in *G*′ and *G*″ for precursors. (G) Hemocompatibility assessment of hydrogel precursors. NS, normal saline. (H and I) Scanning electron microscopy (SEM) micrographs depicting surface architecture and quantitative pore distribution. (J) Compressive modulus of disk-shaped hydrogels. (K and L) Hydrogel stability evaluated through swelling kinetics and enzymatic degradation. Data are presented as mean ± SD (*n* = 3). *P < 0.05, **P < 0.01, and ***P < 0.001 vs. G group; ns, not significant.

In this study, we developed a hydrogel by modifying CQS with methacrylate groups to introduce photocrosslinking capability and enhance its gelation rate and structural stability. As shown in Fig. 2A, CQS-MA could be easily solidified under photocrosslinking. ^1^H nuclear magnetic resonance was also performed for CQS and CQS-MA to verify the presence of methacrylamide groups. When compared to the ^1^H nuclear magnetic resonance spectrum of CQS (Fig. 2B), new signals corresponding to methacryloyl protons emerged in the CQS-MA spectrum between 6 and 5 ppm and at 1.9 ppm. Specifically, the resonances at 5.6 to 5.5 ppm and 5.2 to 5.3 ppm were assigned to the acrylic protons (CH_2_=C(CH_3_)CONH–) of methacrylamide functionalities, while the signal at 1.9 ppm was attributed to methyl protons (CH_2_=C(CH_3_)CO–) in the methacryloyl groups [21]. The incorporation of methacrylate onto the CQS backbone in CQS-MA was further confirmed by FTIR spectroscopy (Fig. S1). Key features in the FTIR spectrum of CQS-MA include the following: a band at 1,630 cm^−1^, indicative of C=O stretching in amide I; a peak at 1,546 cm^−1^, assigned to the N–H bending vibrations of amide II; an absorption at 1,244 cm^−1^, attributed to C–N stretching and N–H bending vibrations in amide III; and a broad band between 3,200 and 3,400 cm^−1^, consistent with N–H and O–H stretching vibrations [22].

Subsequently, we fabricated a ternary composite hydrogel by copolymerizing CQS-MA with GelMA and native type I collagen. The rheological properties of all precursor compositions were evaluated across a range of temperatures. The incorporation of type I collagen and CQS-MA markedly enhanced precursor viscosity (Fig. 2D and E). The temperature dependence of *G*′ and loss modulus (*G*″) was also investigated. The storage modulus (*G*′) of a hydrogel precursor typically decreases with increasing temperature because the material’s viscoelastic behavior prior to crosslinking is governed primarily by reversible, noncovalent interactions and transient chain entanglements. Elevated temperature enhances polymer chain mobility and thermal motion, which weakens hydrogen bonding, ionic interactions, and other physical associations that contribute to elastic energy storage. Notably, the addition of type I collagen and CQS-MA substantially elevated the moduli of GelMA (Fig. 2F and Fig. S2), although gelation continued to occur near 30 °C across all formulations, suggesting minimal disruption to the thermoresponsive gelation behavior of GelMA.

Blood compatibility was assessed for all groups according to ISO 10993-4, a critical standard for evaluating wound dressing materials intended for clinical use. Hemolysis assays were performed to examine erythrocyte integrity following exposure to the precursor solutions. As shown in Fig. 2G, the physiological saline negative control remained transparent, while Triton X-100 (positive control) produced a vivid red supernatant. All precursor compositions yielded clear supernatants, closely resembling the negative control and thus indicating favorable hemocompatibility.

Quantitative hemolysis ratios were determined spectrophotometrically by measuring released hemoglobin. The negative and positive controls were defined as 0% and 100% hemolysis, respectively. The measured hemolysis rates were as follows: G, 2.00% ± 0.02%; G/C, 2.11% ± 0.08%; G/C-CS, 2.05% ± 0.03%; and G/C-CS@FGF-21, 2.19% ± 0.09%. These results conclusively indicate the minimal hemolysis induced by the hydrogels, indicating them as promising candidates for a range of biomedical applications.

The hydrogels were fabricated by photocrosslinking the precursor solutions in a cylindrical mold, resulting in disk-shaped specimens with dimensions of 8 mm in diameter and 2 mm in thickness. Scanning electron microscopy characterization revealed interconnected porous networks across all hydrogel compositions (Fig. 2H). Pore size analysis indicated an average diameter of approximately 150 μm for all groups, with no statistically significant variations observed upon incorporation of type I collagen or CQS-MA (Fig. 2I), suggesting minimal impact on structural porosity.

Mechanical properties were evaluated through compression testing. The compressive moduli were determined as follows: G group, 5.23 ± 0.17 kPa; G/C, 2.46 ± 0.16 kPa; G/C-CS, 10.29 ± 1.54 kPa; and G/C-CS@FGF-21, 12.30 ± 1.11 kPa (Fig. 2J). These results demonstrate that the addition of type I collagen markedly reduced hydrogel stiffness, consistent with earlier findings, whereas CQS-MA inclusion enhanced mechanical strength.

Hydrogel stability was assessed via equilibrium swelling and enzymatic degradation assays (Fig. 2K and L). All compositions remained structurally stable throughout swelling experiments, indicating that despite the mechanical softening induced by collagen, the hydrogels retained sufficient integrity for subsequent cell culture applications. Notably, under high-trypsin conditions that mimic a diabetic wound environment, all hydrogels displayed a strategically designed, rapid disintegration within 80 min, thereby facilitating clearance as foreign materials and enabling the release of encapsulated bioactive factors. Most importantly, the incorporation of QCS-MA significantly attenuated the degradation rate in this challenging microenvironment, underscoring its role in enhancing the hydrogel’s durability. This tunable degradation behavior highlights the material’s adaptive potential for stage-specific wound-healing applications [23].

### The G/C hydrogel better promoted fibroblast growth

**Fig. 3.**
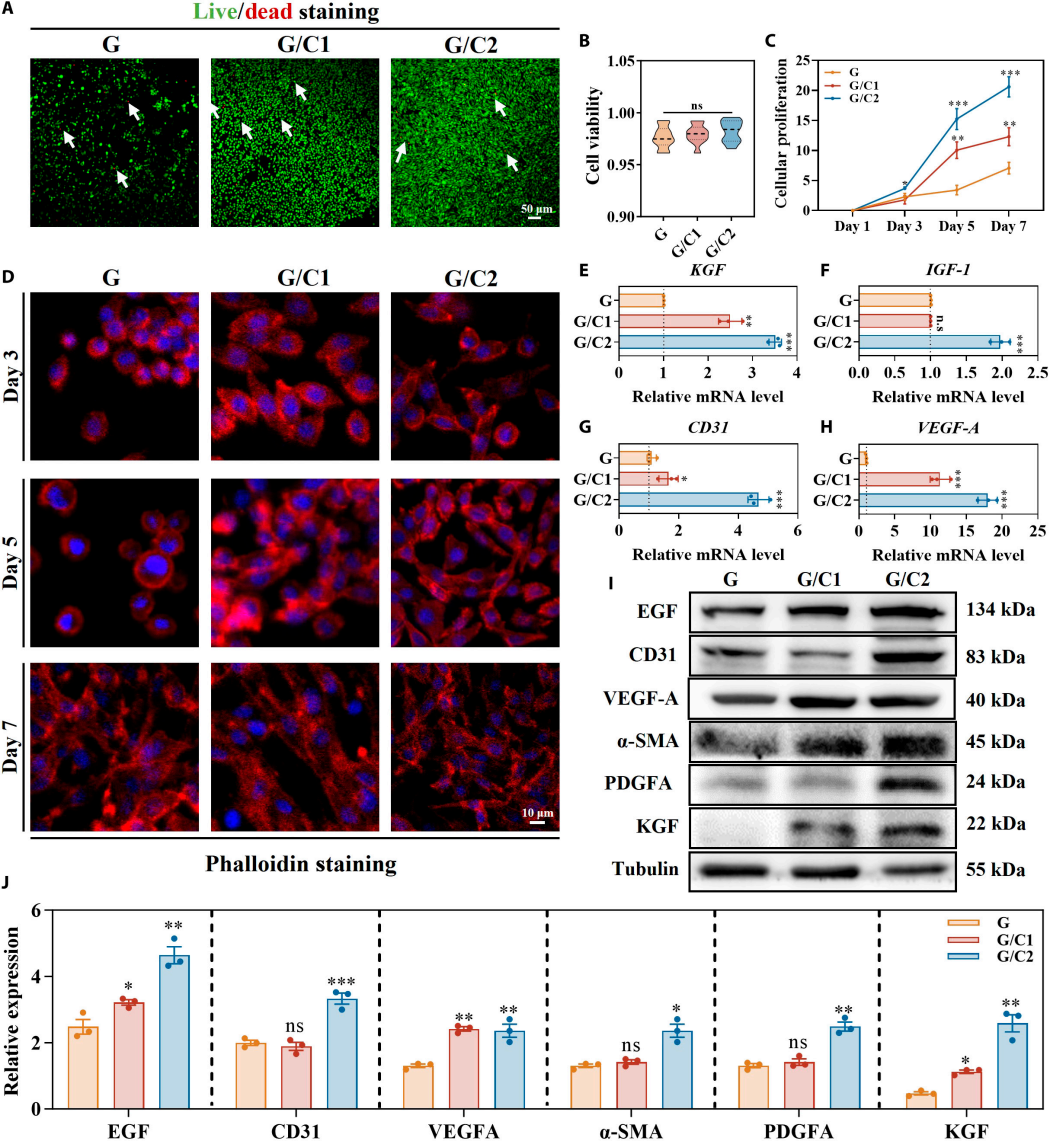
Biological analysis of L929 cells on the G/C hydrogel surface. (A and B) Live/dead assay and quantification of L929 cells on G and G/C hydrogel surfaces. (C) Cellular proliferation rates of L929 cells. (D) Cellular morphology of L929 cells at various time points. (E to H) Quantitative real-time polymerase chain reaction (qRT-PCR) of wound-healing-related gene expressions. (I and J) Western blotting and quantification analysis of wound-healing-related proteins. Data are presented as mean ± SD (*n* = 3). *P < 0.05, **P < 0.01, and ***P < 0.001 vs. G group; ns, not significant. mRNA, messenger RNA; IGF-1, insulin-like growth factor 1; α-SMA, alpha smooth muscle actin; PDGFA, platelet-derived growth factor subunit A.

Being the predominant protein in the human body, collagen is indispensable in wound healing, serving as both a physical scaffold and a bioactive regulator that guides each stage from hemostasis through remodeling. To enhance bioactivity, we incorporated varying concentrations of pure collagen into GelMA, and cultured L929 mouse fibroblasts on its surface for biological analysis. To assess the cytocompatibility of the hydrogels, a live/dead staining assay was performed. In this method, viable cells fluoresced green, while nonviable cells were labeled red, with white arrows used to highlight deceased cells (Fig. 3A). Minimal cell death was observed across all hydrogel surfaces, a finding further supported by quantitative evaluation (Fig. 3B), which revealed that the mortality rate of L929 cells remained below 3% for every hydrogel formulation. Additionally, the proliferative activity of L929 cells was examined using CCK-8 assays at time points of 1, 3, 5, and 7 d. All hydrogel compositions successfully supported cell growth, while collagen-containing hydrogels, particularly the G/C2 group, markedly accelerated proliferation (Fig. 3C). A similar trend was observed in collagen-containing electrospun scaffolds, where the presence of collagen also promoted fibroblast proliferation [24]. This effect could be attributed to the presence of arginine-glycine-aspartic acid (RGD) sequences in collagen, which facilitate cell adhesion and growth [25]. Phalloidin (Fig. 3D) and integrin beta 1 staining (Fig. S3) also corroborated these findings, where the L929 cells on pure GelMA remained largely rounded through day 5, elongating only by day 7, whereas those on G/C1 and G/C2 exhibited prominent elongation as early as day 3, highlighting collagen’s role in facilitating cellular adhesion. Gene and protein expressions were analyzed for the KGF, insulin-like growth factor 1 (IGF-1), VEGF-A, and CD31 markers in L929 cells cultured on hydrogels containing varying concentrations of type I collagen. KGF and IGF-1, which drive epithelial proliferation and reepithelialization, and VEGF-A and CD31, key regulators of angiogenesis and vascular remodeling [26], were all significantly upregulated in collagen-supplemented hydrogel compared to those in controls, with the G/C2 formulation showing the greatest induction (Fig. 3E to J and Fig. S4a and b). These results align with previous studies demonstrating that collagen-rich environments influence fibroblast gene expression through direct cell–matrix interactions and integrin-mediated signaling [27]. Concordantly, Western blotting and immunofluorescence analyses revealed that G/C2-cultured cells exhibited the highest protein levels of EGF, α-SMA, and PDGFA, further validating the superior bioactivity of the G/C2 hydrogel and justifying its selection for subsequent experiments (Fig. 3I and J).

### FGF-21 manipulated macrophage differentiation and fibroblast migration

**Fig. 4.**
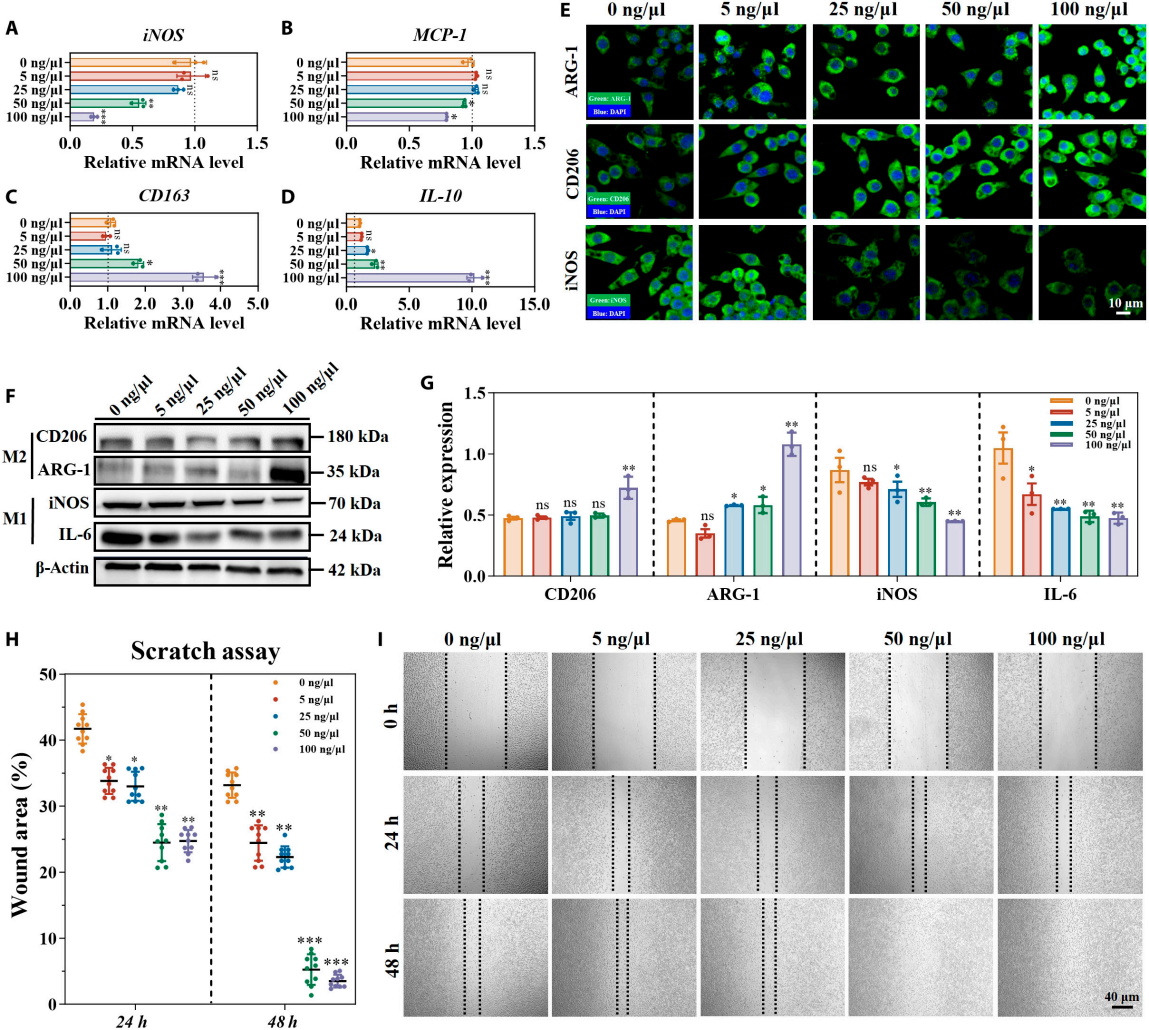
Biological analysis of RAW264.7 and L929 cells stimulated with various concentrations of FGF-21. (A to D) qRT-PCR of M1- and M2-related gene expressions in RAW264.7 cells. (E) Immunofluorescence staining of M1- and M2-related proteins in RAW264.7 cells. (F and G) Western blotting and quantification analysis of M1- and M2-related protein expressions in RAW264.7 cells. (H and I) Scratch assay and quantification analysis of L929 cells stimulated with various concentrations of FGF-21. Data are presented as mean ± SD (*n* = 3). *P < 0.05, **P < 0.01, and ***P < 0.001 vs. G group; ns, not significant. MCP-1, monocyte chemoattractant protein-1; DAPI, 4′,6-diamidino-2-phenylindole.

FGF-21 is an endocrine hormone that regulates glucose and lipid metabolism and is primarily secreted by the liver, enhances insulin sensitivity, and modulates energy homeostasis. In diabetic wound healing, FGF-21 could accelerate tissue repair by attenuating inflammation, promoting angiogenesis, and stimulating fibroblast proliferation, making it a promising therapeutic factor [28]. To identify the optimal FGF-21 dose for macrophage modulation, RAW264.7 cells were treated with 0, 5, 25, 50, and 100 ng/μl FGF-21. To analyze the expression of genes associated with macrophage differentiation, including iNOS and monocyte chemoattractant protein-1 (MCP-1) for M1 polarization and CD163 and IL-10 for M2 polarization, qRT-PCR was employed. RAW264.7 cells exposed to 100 ng/μl FGF-21 exhibited the greatest upregulation of M2 genes and down-regulation of M1 genes (Fig. 4A to D). Western blotting and immunofluorescence corroborated these findings, showing increased CD206 and ARG-1 and decreased iNOS and IL-6 at 100 ng/μl (Fig. 4E to G and Fig. S5a). These findings suggest that FGF-21 promotes a phenotypic shift in macrophages from the pro-inflammatory M1 state toward the anti-inflammatory M2 phenotype. This observation is consistent with prior research demonstrating that FGF-21 substantially suppresses inflammatory cytokine expression macrophages stimulated by lipopolysaccharide (LPS) through Nrf2–nuclear factor-κB (NF-κB) signaling axis [29].

The effect of FGF-21 on L929 cell migration and gene expression was also evaluated. In the scratch assay, treatment with 100 ng/μl FGF-21 achieved complete wound closure within 48 h, followed by near-complete closure at 50 and 25 ng/μl (Fig. 4H), as confirmed by quantitative analysis using ImageJ normalized to the initial scratch area (Fig. 4I). qRT-PCR further revealed that 100 ng/μl FGF-21 elicited the greatest upregulation of skin-regenerative genes, such as EGF, KGF, and IGF-1, and concurrently suppressed the inflammatory mediator MMP-9 (Fig. S5b to e). These effects likely result from FGF-21 induced activation of AKT and c-Jun N-terminal kinase (JNK) signaling pathways in fibroblasts [30]. Collectively, these data demonstrate that 100 ng/μl FGF-21 most effectively promotes M2 macrophage polarization and enhances fibroblast function, justifying its selection for subsequent in vitro and in vivo studies.

### Distinct transcriptional profile of macrophages on composite hydrogels

Based on the aforementioned results, we subsequently incorporated CQS-MA into the G/C2 hydrogel, along with 100 ng/μl of FGF-21, to evaluate the effects of CQS MA on RAW264.7 cellular behavior and its influence on FGF-21 release post-encapsulation.

**Fig. 5.**
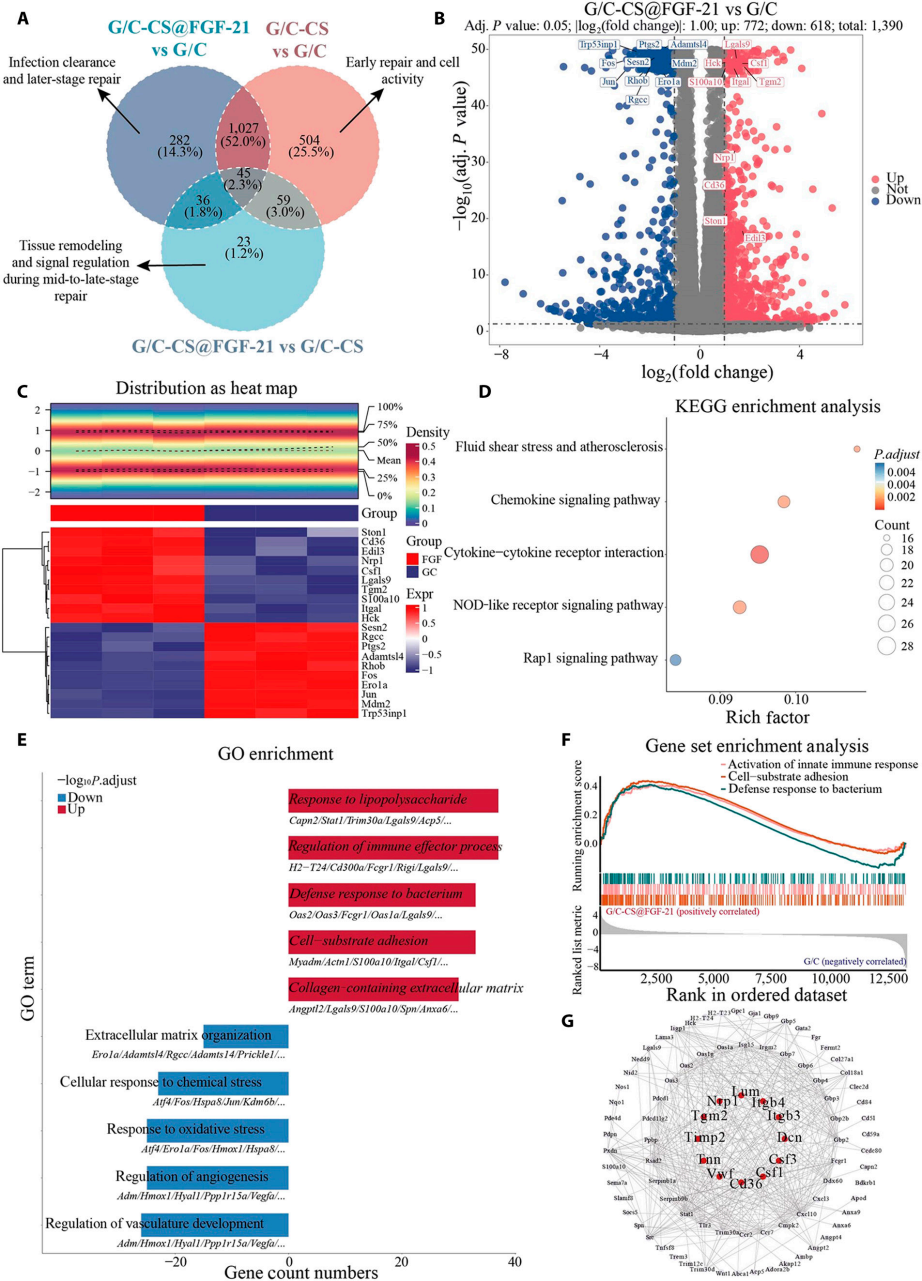
Transcriptional profiles for RAW264.7 cells on hydrogels. (A) Statistics of differently expressed genes between all 3 compositions of hydrogels. (B) Volcano plot transcriptional landscape between G/C and G/C-CS@FGF-21. The *x*-axis represents to the log_2_-transformed fold change, while the *y*-axis corresponds to the negative logarithm of the *P* value following adjustment for multiple comparisons. (C) The heat map of wound-healing- and inflammation-related genes between G/C and G/C-CS@FGF-21. (D) Kyoto Encyclopedia of Genes and Genomes (KEGG) enrichment analysis between G/C and G/C-CS@FGF-21. (E) Gene Ontology (GO) enrichment analysis between G/C and G/C-CS@FGF-21. (F) Gene set enrichment analysis (GSEA) between G/C and G/C-CS@FGF-21. (G) Protein–protein interaction networks of differentially expressed proteins between G/C and G/C-CS@FGF-21.

To investigate the impact of CQS-MA and FGF-21 on the transcriptional signature of macrophages, global transcriptomic analysis of RAW264.7 cells grown on G/C, G/C-CS, and G/C-CS@FGF-21 substrates was carried out using RNA-seq (Fig. 5 and Figs. S6 and S7). Principal component analysis demonstrated a markedly distinct transcriptional profile in RAW264.7 cells treated with CQS-MA and FGF-21 relative to that in control samples (Fig. 5A). Notably, key regulators of proliferation and migration, fibroblast growth factor receptor 1 (FGFR1), integrin subunit alpha L​ (ITGAL), and interleukin 17 receptor A (IL17RA), were significantly upregulated in the G/C-CS@FGF-21 group compared with those in G/C (Fig. 5B). ITGAL encodes the α-subunit of lymphocyte function-associated antigen 1 (LFA-1), facilitating macrophage adhesion to endothelial ​intercellular adhesion molecule-1 (ICAM-1) and subsequent transendothelial migration [31], while IL17RA is critical for IL-17-mediated signaling and promotes a reparative M2-like phenotype [32]. Conversely, the expression of inflammatory mediators, including hyaluronidase 1 (HYAL1), allograft inflammatory factor 4 (AIF4), and adrenomedullin (ADM), was markedly down-regulated. ADM is a vasoactive peptide that macrophages produce in response to inflammatory stimuli. Down-regulated expression of ADM could decrease the secretion of IL-6 and IL-1β from both nonstimulated and LPS-stimulated macrophages, suggesting that ADM plays important roles in the inflammatory response [33]. HYAL1 is a lysosomal enzyme that degrades high-molecular-weight hyaluronic acid (HA) into oligosaccharides, regulating ECM turnover and inflammatory signaling in macrophages [34]. Decreased HYAL1 expression in RAW264.7 cells reduces HA fragmentation, preserving a pericellular HA-rich matrix that promotes fibroblast and keratinocyte migration into the wound bed and maintains hydration conducive to healing [35].

Kyoto Encyclopedia of Genes and Genomes pathway enrichment analysis was conducted using standard gene set enrichment methods to identify overrepresented signaling cascades on the G/C-CS@FGF-21 group compared with those on G/C (Fig. 5D). Pathways associated with inflammation control and immune modulation, particularly the chemokine signaling route and cytokine–cytokine receptor interaction, were prominently enriched. Wound-repair-related mechanisms, including NOD-like receptor (NLR) signaling and ​Ras-associated protein 1 (Rap1) transduction pathways, also displayed substantial overrepresentation. The NLR pathway, notably via the ​NLR family pyrin domain containing 3 (NLRP3) inflammasome, governs the early inflammatory phase by detecting damage-associated molecular patterns, activating caspase-1, and promoting IL-18 and IL-1β maturation to recruit immune cells and initiate tissue repair [36]. Conversely, Rap1, a small GTPase, orchestrates cytoskeletal reorganization and cell–cell junction dynamics through effectors like ephexin and Rho1, thereby facilitating collective cell migration and epithelial gap closure [37]. Together, these pathways coordinate the inflammatory to proliferative transition, ensuring timely reepithelialization, angiogenesis, and ECM deposition. Gene Ontology enrichment analysis (Fig. 5E) of RNA-seq data comparing the G/C-CS@FGF-21 group with G/C revealed that upregulated genes were chiefly enriched in immune regulatory and ECM organization processes, including regulation of immune effector processes, defense response to bacterium, cell–substrate adhesion, and collagen-containing ECM. Conversely, down-regulated genes were primarily associated with cellular responses to chemical stress and oxidative stress responses. Gene set enrichment analysis (Fig. 5F) across the entire transcriptome further demonstrated significant enrichment of innate immune activation, cell–matrix adhesion, and bacterial defense pathways under FGF-21 stimulation, indicating its role in activating immune responses and tissue repair mechanisms. The heat map of DEGs revealed that FGF-21 markedly upregulates STON1, CD36, EDIL3, and other mediators of tissue repair and immune regulation (Fig. 5C), while concomitantly down-regulating ERO1A, JUN, and MDM2, genes that associated with metabolic activation and stress responses. Collectively, these transcriptional alterations underscore FGF-21’s efficacy in promoting wound-healing processes.

### The G/C-CS@FGF-21 hydrogel promoted macrophage differentiation

**Fig. 6.**
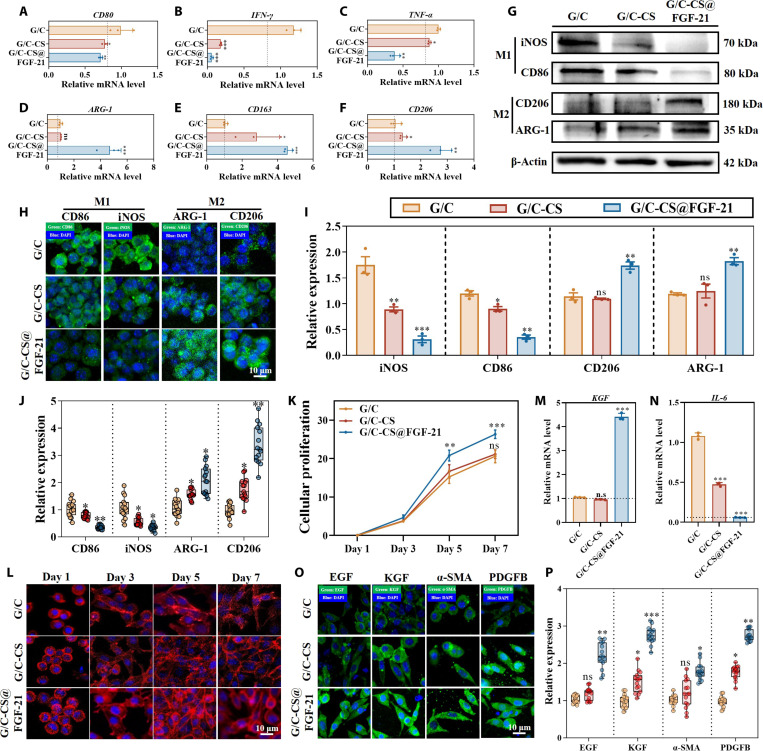
Biological analysis of RAW264.7 and L929 cells grown on different hydrogel formulations. (A to F) qRT-PCR of M1- and M2-associated gene expressions in RAW264.7 cells. (G) Western blotting of M1- and M2-associated proteins in RAW264.7 cells. (H) Immunofluorescence staining of M1- and M2- associated proteins in RAW264.7 cells. (I) Quantification analysis of Western blotting of M1- and M2-associated proteins of RAW264.7 cells. (J) Quantification analysis of immunofluorescence staining of M1- and M2-associated proteins of RAW264.7 cells. (K) Cellular proliferation rates of L929 cells. (L) Cellular morphology of L929 cells at various time point. (M and N) Quantification of wound-healing-associated gene expression in L929 cells via qRT-PCR. (O and P) Immunofluorescence staining and quantification of wound-healing-related proteins detected in L929 fibroblasts. Data are presented as mean ± SD (*n* = 3). *P < 0.05, **P < 0.01, and ***P < 0.001 vs. G group; ns, not significant. PDGFB, platelet-derived growth factor subunit B.

The release of FGF-21 was analyzed with enzyme-linked immunosorbent assay (Fig. S8), where FGF-21 release peaked at day 1, declined subsequently, and was nearly complete by day 10. For RAW264.7 cells cultured on all groups of hydrogels, we further analyzed the gene expression of M1 genes (CD80, TNF-α, and IFN-γ) and M2 genes (CD206, ARG-1, and CD163). Incorporation of CQS-MA significantly reduced M1-associated genes expression while elevating M2-associated genes, suggesting a shift toward the reparative M2 phenotype; this effect was further potentiated by FGF-21, confirming that composite hydrogel encapsulation preserves FGF-21 bioactivity (Fig. 6A to F). The parallel protein expressions of M1-related markers (iNOS and CD86) and M2-related markers (CD206 and ARG-1) were analyzed with Western blotting (Fig. 6G and I) and immunofluorescence staining (Fig. 6H and J); they likewise demonstrated enhanced M2 polarization with CQS-MA incorporation and additional augmentation by FGF-21. These results corroborate prior studies showing that CQS, alone or in combination with other biomaterials or ions, promotes M2-type macrophage polarization via the cGAS–STING, PPARγ, and NLRP3 signaling pathways, consistent with known chitosan-mediated mechanisms [38].

For L929 cells, to evaluate the proliferation rate on various hydrogel compositions, the CCK-8 assay was employed, with the G/C group serving as the control group (Fig. 6K). Results indicated that the incorporation of CQS-MA had negligible impact on L929 cell growth, whereas FGF-21 demonstrated the ability to enhance cellular proliferation. CQS-MA is water-soluble across a broad pH range due to its permanent quaternary ammonium groups and, owing to its inherent cationic nature, facilitates favorable electrostatic engagement with negatively charged cellular membranes [39]. L929 cellular morphology on hydrogel surfaces assessed by phalloidin staining (Fig. 6L) revealed that cells on all hydrogel formulations adopted elongated, spindle-shaped phenotypes by day 3, indicating that CQS-MA supports robust cell-adhesive properties. qRT-PCR and immunofluorescence analyses further showed synergistic upregulation of EGF, KGF, and α-SMA, alongside down-regulation of the pro-inflammatory cytokine IL-6, highlighting cooperative interactions between CQS-MA and FGF-21 in promoting wound-healing phenotypes (Fig. 6M to P). These findings align with previous reports in which CQS-containing dressings enhanced fibroblast migration, increased the expression of pro-healing factors such as PDGFs, and suppressed IL-6 production [40]. In summary, the G/C-CS@FGF-21 composite hydrogel exhibited potent bioactivity in both fibroblasts and macrophages, justifying its advancement to in vivo evaluation.

### The G/C-CS@FGF-21 hydrogel promoted skin regeneration on diabetic mice

**Fig. 7.**
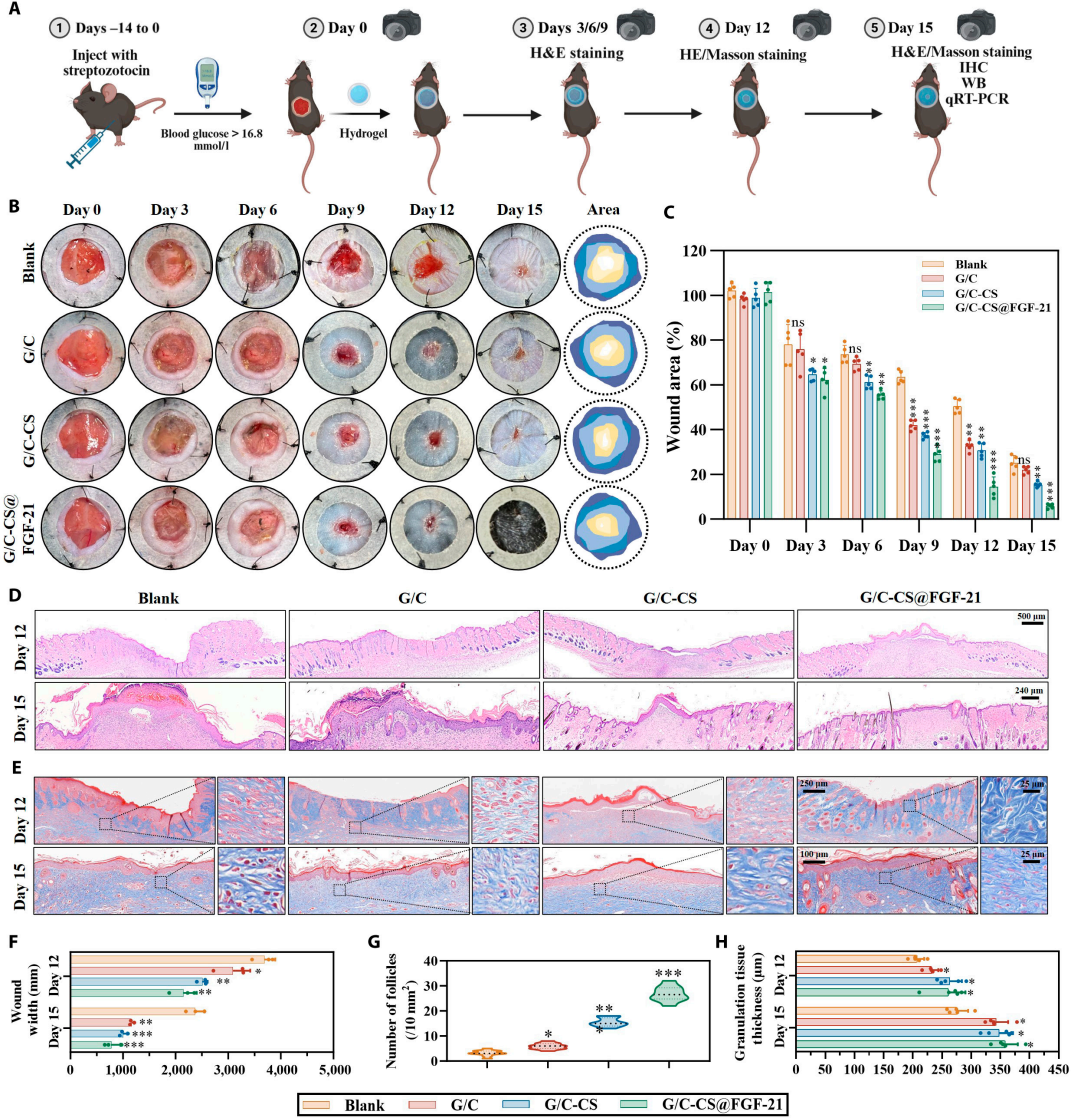
In vivo evaluation of FGF-21-loaded hydrogel in a full-thickness wound-healing model. (A) Schematic diagram of the experimental timeline and treatment protocol for wound healing in a diabetic mouse model. (B) Representative photographic documentation and diagrammatic overview of wound closure from days 0 to 15. (C) Quantified wound closure rates over the 15-d observation period. (D) Histological analysis via hematoxylin and eosin (H&E) staining on days 12 and 15. (E) Collagen deposition assessed by Masson’s trichrome staining on days 12 and 15. Morphometric evaluation of (F) wound width, (G) hair follicle density, and (H) granulation tissue thickness. Data are presented as mean ± SD (*n* = 3). *P < 0.05, **P < 0.01, and ***P < 0.001 vs. G group; ns, not significant. IHC, immunohistochemistry; WB, Western blotting.

To assess the therapeutic potential of G/C, G/C-CS, and G/C-CS@FGF-21 hydrogels in diabetic wound healing, a full-thickness skin injury model was established in diabetic mice (Fig. 7B). Wounds in the blank control group were covered with a VSD membrane and received no further treatment throughout the 15-d healing period. In contrast, experimental groups were administered the corresponding hydrogels before identical VSD dressing application. All skin defects were created uniformly in size on day 0. Wound closure progression was documented photographically for all groups on days 0, 3, 6, 9, 12, and 15 (Fig. 7B).

Prior to day 6, the wound areas in the G/C-CS and G/C-CS@FGF-21 groups were similar, while they were slightly smaller than those of the G/C and blank groups. From days 9 to 15, the G/C-CS@FGF-21 group exhibited significantly accelerated wound closure compared with all other treatments, culminating in complete healing by day 15. These findings were further confirmed by quantitative wound closure analysis using ImageJ, where the wound closure rates were 24.99% ± 3.46%, 21.87% ± 1.64%, 15.44% ± 1.23%, and 5.83% ± 0.99% for the blank, G/C, G/C-CS, and G/C-CS@FGF-21 groups on day 15, respectively (Fig. 7C).

On days 6, 9, 12, and 15, regenerated skin tissues were collected for H&E staining and quantitative analysis (Fig. 7D and Fig. S9a). Wound-width measurements demonstrated (Fig. 7E) that from day 6 onward, the G/C-CS@FGF-21 group exhibited significantly narrower wounds than controls, achieving near-complete closure by day 15. Hair follicle regeneration is a key indicator of skin repair, which are highly conserved sensory structures that not only mediate immune defenses against pathogens but also play essential roles in angiogenesis, neurogenesis, and the wound-healing process [41]. Hair follicle quantification was performed manually using H&E-stained sections. The G/C-CS@FGF-21 group exhibited a significantly greater number of hair follicles compared to all other groups, followed by the G/C-CS, G/C, and blank groups (Fig. 7F), suggesting the promotive role of FGF-21 in follicular regeneration. Granulation tissue thickness is widely regarded as a fundamental indicator of wound-healing progression. As illustrated in Fig. 7G, all experimental groups displayed comparable thicknesses of granulation tissue on days 12 and 15, which were significantly greater than those in the blank group. This implies that collagen incorporation served as the primary factor contributing to the restoration of granulation tissue thickness [42].

**Fig. 8.**
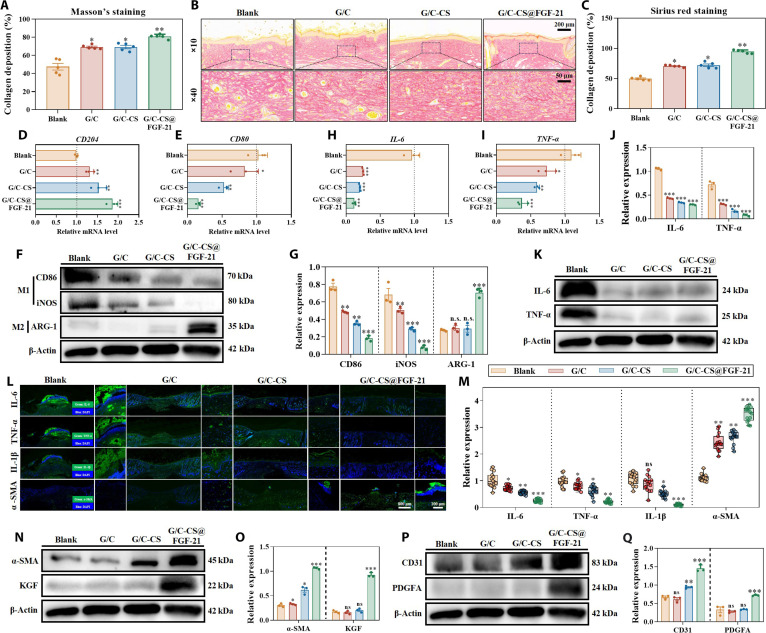
Molecular expression profiles in wound-healing tissues. (A) Quantification of Masson’s staining. (B and C) Sirius Red staining and quantification. (D to G) Gene and protein expression of M1- and M2-related markers. (H to K) Gene and protein expression of inflammation-related markers. (L and M) Immunofluorescence staining and quantification of wound-healing-related markers. (N and O) Western blotting and quantification of skin regeneration markers. (P and Q) Western blotting and quantification of angiogenesis-related markers. Data are presented as mean ± SD (*n* = 3). *P < 0.05, **P < 0.01, and ***P < 0.001 vs. G group; ns, not significant.

During the maturation stage of wound healing, collagen accumulation is crucial for scar tissue development, wound contraction, and the recovery of mechanical strength via crosslinking interactions between collagen and fibroblasts [25]. Masson’s trichrome staining at days 12 and 15 (Figs. 7D and 8A) revealed significantly greater collagen accumulation in the G/C-CS@FGF-21 group compared to those of all other treatments, with no appreciable difference between the G/C and G/C-CS groups. Similar results were also found in Sirius Red staining, where the red areas represented collagen fibers, while yellow areas indicated muscle fibers and blood cells. The G/C-CS@FGF-21 group showed deeper red collagen fibers that were more organized, suggesting a higher collagen content and a more mature tissue structure, which were further verified with quantitative analysis (Fig. 8B and C). These findings suggest that FGF-21 enhances collagen deposition during wound healing, potentially due to the ability of the FGF family to activate downstream ERK1/2 and JNK signaling pathways via the FGFR–β-Klotho receptor complex [43]. Consistent with this hypothesis, previous studies have shown that JNK activation not only promotes fibroblast migration but also directly upregulates the transcription and synthesis of fibrillar collagens [20].

On day 15, regenerated skin specimens were collected and evaluated using qRT-PCR, immunofluorescence staining, and Western blot to examine markers associated with inflammation, macrophage polarization, skin renewal, and angiogenesis. Regarding macrophage polarization, the G/C-CS group facilitated a shift from a pro-inflammatory (M1) to an anti-inflammatory (M2) phenotype, evidenced by reduced levels of CD80, CD86, and iNOS, in conjunction with increased expression of CD204 and ARG-1 (Fig. 8D to G). Chitosan modulates Toll-like receptors and the NLRP3 inflammasome in a manner influenced by both concentration and formulation, leading to inhibition of pro-inflammatory cytokines and enhanced secretion of transforming growth factor-β (TGF-β) and IL-10 [44]. By attenuating NF-κB activation and enhancing STAT6 phosphorylation, chitosan could also upregulate M2-associated genes such as ARG-1 and CD206, leading to sustained M2 marker expression [45]. This M2 bias was amplified in the G/C-CS@FGF-21 group, in which FGF-21, via binding to the FGFR1c–β-Klotho receptor complex, inhibits NF-κB and CHOP-mediated apoptosis and attenuates LPS-induced M1 marker expression through AMP-activated protein kinase (AMPK) pathway activation [46]. AMPK signaling also enhances macrophage autophagic flux to stabilize the M2 phenotype [47]. Collectively, these data confirm that CQS-MA promotes M2 polarization and that FGF-21 further reprograms macrophages to resolve inflammation and support tissue repair.

Pro-inflammatory markers, such as IL-6 and TNF-α, were analyzed (Fig. 8H to M), showing that the inclusion of CQS-MA and FGF-21 can relatively reduce the inflammation process. Chitosan inhibits inflammation through a combination of receptor-mediated signaling, suppression of pro-inflammatory pathways, antioxidant activity, and physical barrier effects. Chitosan has been demonstrated to suppress pro-inflammatory cytokines, including IL-12, IL-6, and TNF-α, and concurrently enhance the secretion of anti-inflammatory factors such as IL-10 and TGF-β according to several studies [48]. FGF-21 has been shown to inhibit the secretions of MCP-1, IL-6, and TNF-α in adipocytes, hepatocytes, and macrophages while increasing TGF-β and IL-10 levels, creating an anti-inflammatory milieu favorable for tissue repair [48].

In addition, markers like TGF-β1, α-SMA, and KGF (Fig. 8L to O and Fig. S9b) that correlated with skin regeneration were significantly upregulated compared with those in the control group, suggesting that CS and FGF-21 combination groups could promote diabetic wound healing. The expressions of PDGFA and CD31 were also analyzed and demonstrated that FGF-21 could enhance the angiogenesis process during wound healing (Fig. 8P and Q and Fig. S9c). Consistent with these findings, FGF-21 enhanced both migratory activity and tube formation capability in human brain microvascular endothelial cells [19]. In addition, FGF-21 could enhance the microvessel density to levels similar to that of VEGF treatment in wound healing [49].

## Conclusion

In this study, we engineered a multifunctional, photocrosslinkable hydrogel by CQS-MA and copolymerizing it with GelMA and type I collagen, into which FGF-21 was in situ entrapped. In vitro, the G/C-CS@FGF-21 hydrogel robustly drove the phenotypic shift of macrophages from M1 toward M2; enhanced fibroblast proliferation, migration, and expression of key wound-healing genes; and supported angiogenic signaling. For an in vivo diabetic mouse model, treatment with the G/C-CS@FGF-21 hydrogel markedly accelerated wound closure; improved granulation tissue formation, collagen deposition, and hair follicle regeneration; and modulated local inflammatory and regenerative markers to favor tissue repair. Collectively, these findings demonstrate that the G/C-CS@FGF-21 hydrogel offers a synergistic strategy to promote all stages of diabetic wound repair, which demonstrates considerable translational potential in medical settings.

## Data Availability

All relevant data supporting the findings of this research can be obtained by contacting the corresponding authors.
