# Supplementary material for: Photocrosslinkable Chitosan Quaternary Ammonium Salt-Based Ternary Hydrogel with Fibroblast Growth Factor 21 for Diabetic Wound Healing
Source: Biomater Res. 2026 Jan 27;30:0309. doi: 10.34133/bmr.0309 (PMC12835494; doi:10.34133/bmr.0309)
Supplement: Supplementary 1 — Figs. S1 to S9 [file bmr.0309.f1.docx]

**Photocrosslinkable Chitosan‑Quaternary Ammonium Salt Based Ternary Hydrogel with FGF‑21 for Diabetic Wounds Healing**

Jingying Hu^1, 2, #^, Yongqi Xu^2, #^, Danni Zhou ^2, 3^, Kaixuan Chen ^2, 3^, Jinwen Jiang^2, 3^, Min Lin^1, 2^, Wenjie Chen^1, 2^, Jing Wu^2, 3^, Hongde Jiang^2, 3^, Mengxiang Zhu^3^, Bin Zhang^4, *^, Kailei Xu^3, *^, Peng Wei^1, *^

^1^Department of Plastic Surgery, The Affiliated People's Hospital of Ningbo University, Ningbo, China, 315040

^2^Health Science Center, Ningbo University, Ningbo 315211, China

^3^Center for Medical and Engineering Innovation, Central Laboratory, the First Affiliated Hospital of Ningbo University, Ningbo, Zhejiang 315010, China

^4^Department of Hand and Foot Microsurgery, Yuyao People Hospital, Yuyao, Zhejiang 315400, China

^#^These authors contributed equally to this work.

^*^Corresponding authors: weipeng@nbu.edu.cn; xukailei@zju.edu.cn; bzsweetin@163.com


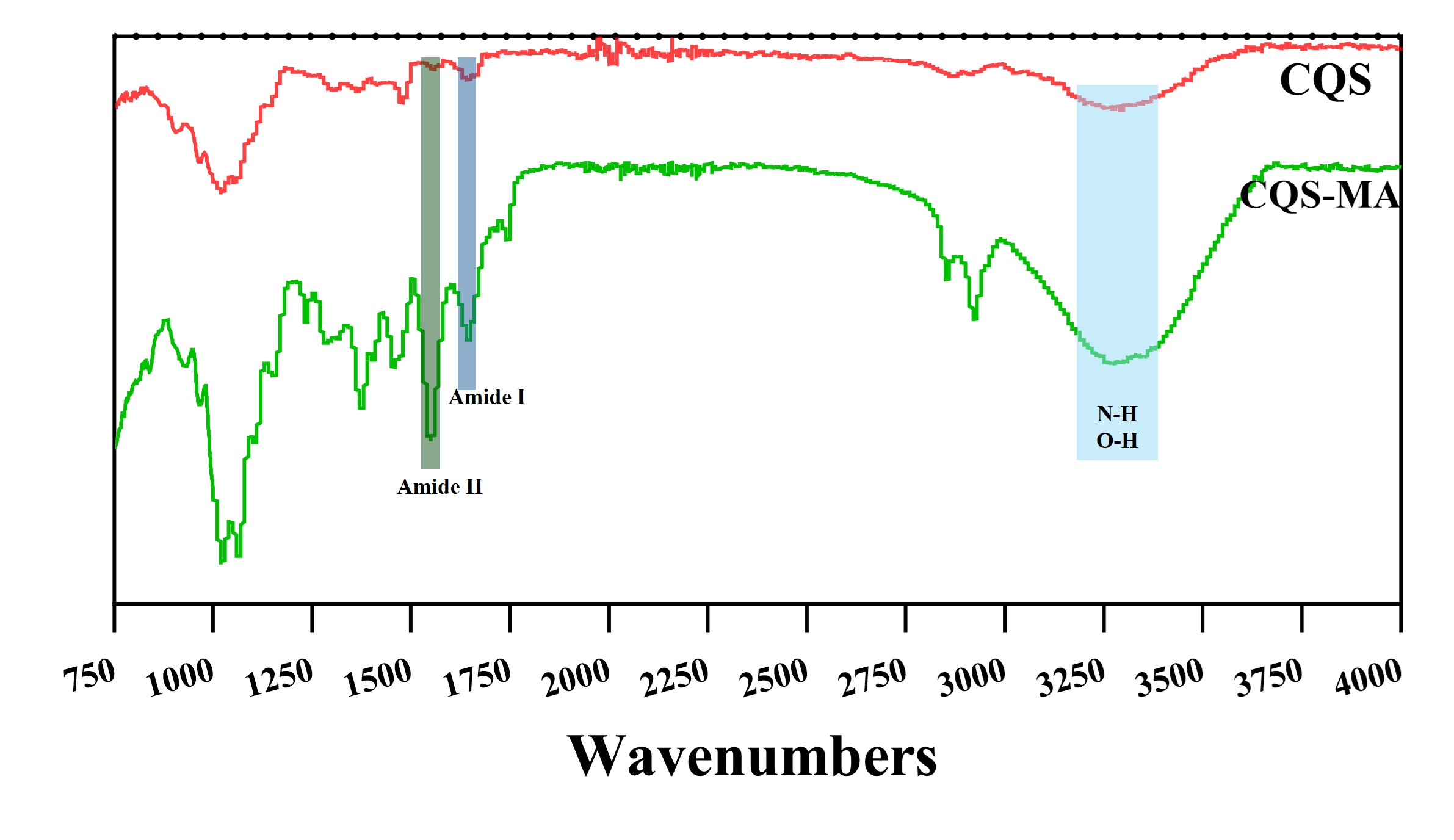


Figure S1. The FTIR analysis of QCS and QCS-MA.


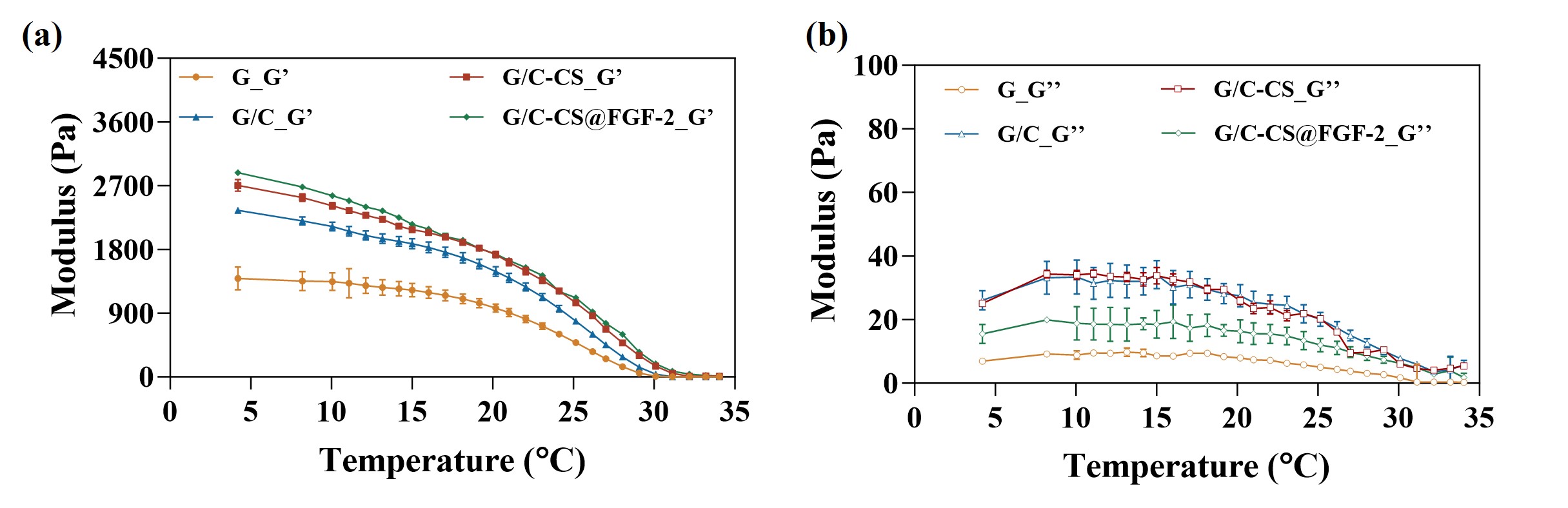


Figure S2. (a) Thermal variations in storage modules (G’) for precursors. (b) Thermal variations in loss modules (G’’) for precursors.


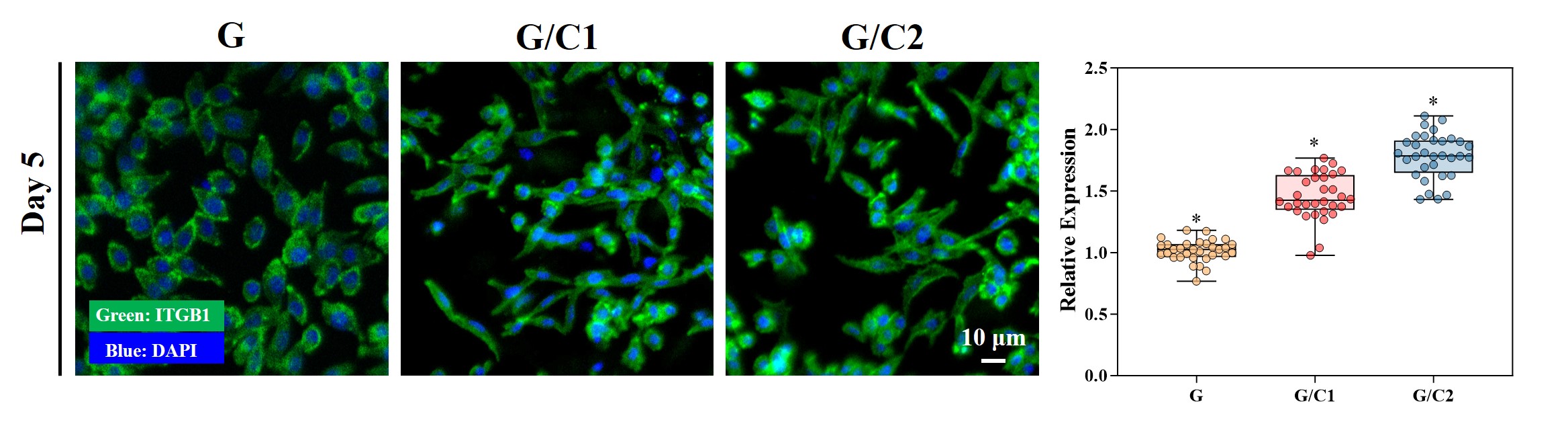


Figure S3. Immunofluorescence staining of L929 cells to characterize cell adhesion capability. Nuclei were stained with DAPI (blue), and integrin β1 markers were stained with FITC (green).


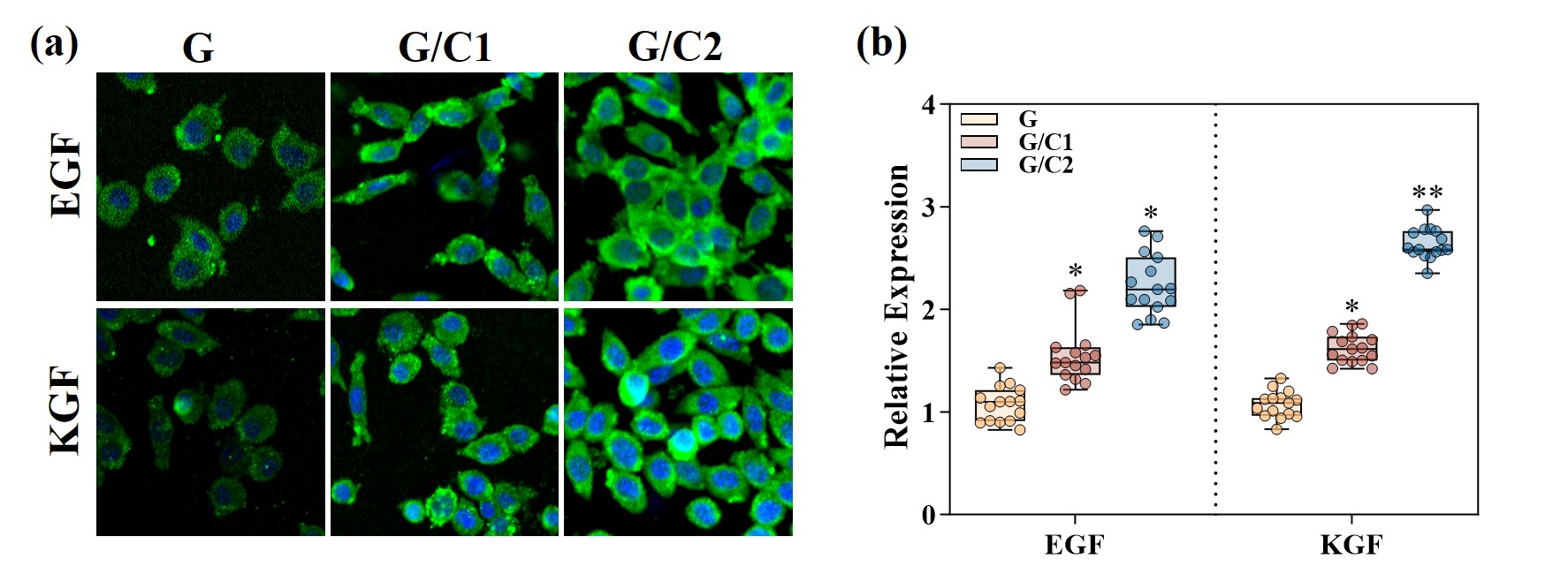


Figure S4. The evaluation of EGF and KGF protein expression for L929 cells on G, G/C1, and G/C1. (a) The immunofluorescence staining of EGF and KGF; (b) Quantification analysis of immunofluorescence staining.


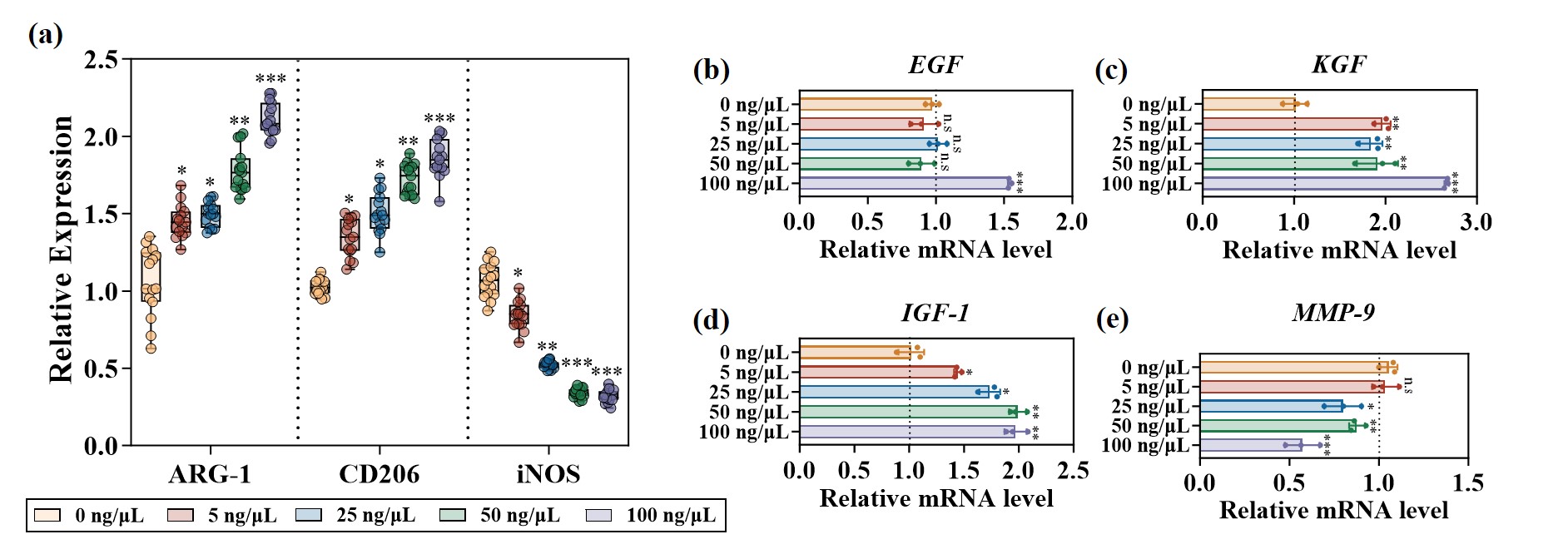


Figure S5. (a) Quantification analysis of immunofluorescence staining for ARG-1, CD206, and iNOS in RAW264.7 cells; (b) qRT-PCR results of EGF, KGF, IGF-1, and MMP-9 gene expressions in L929 cells.


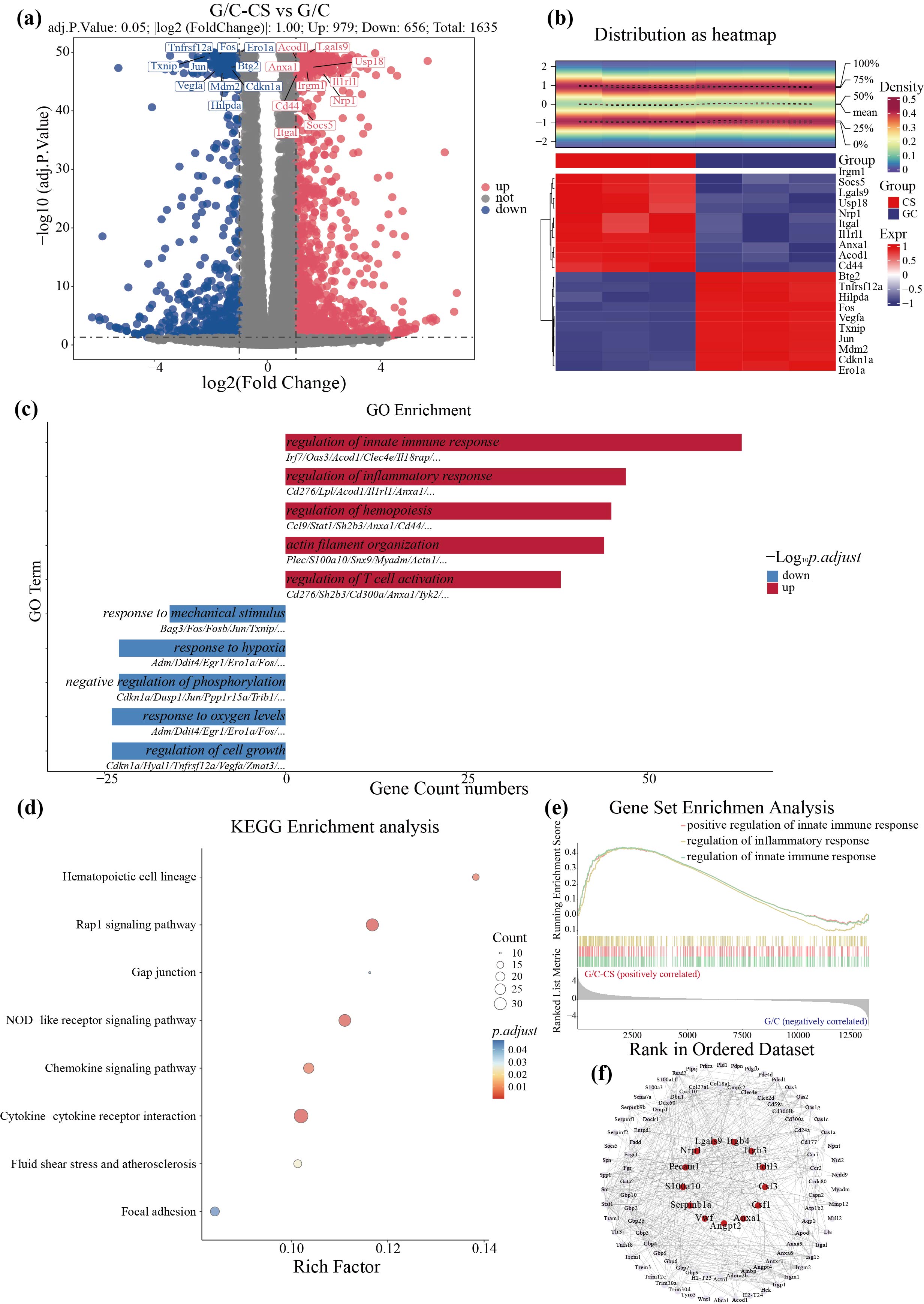


Figure S6. Transcriptional profiles for RAW264.7 cells on G/C and G/C-CS hydrogels. (a) Volcano plot transcriptional landscape between G/C and G/C-CS. The x-axis represents log2 transformed fold change, and the y-axis shows the log transformed p value adjusted for multiple test correction; (b) The heat map of wound healing and inflammation related genes between G/C and G/C-CS; (c) GO enrichment analysis between G/C and G/C-CS; (d) KEGG enrichment analysis between G/C and G/C-CS; (f) Gene set enrichment analysis between G/C and G/C-CS; (g) Protein-protein interaction networks of differentially expressed proteins between G/C and G/C-CS.


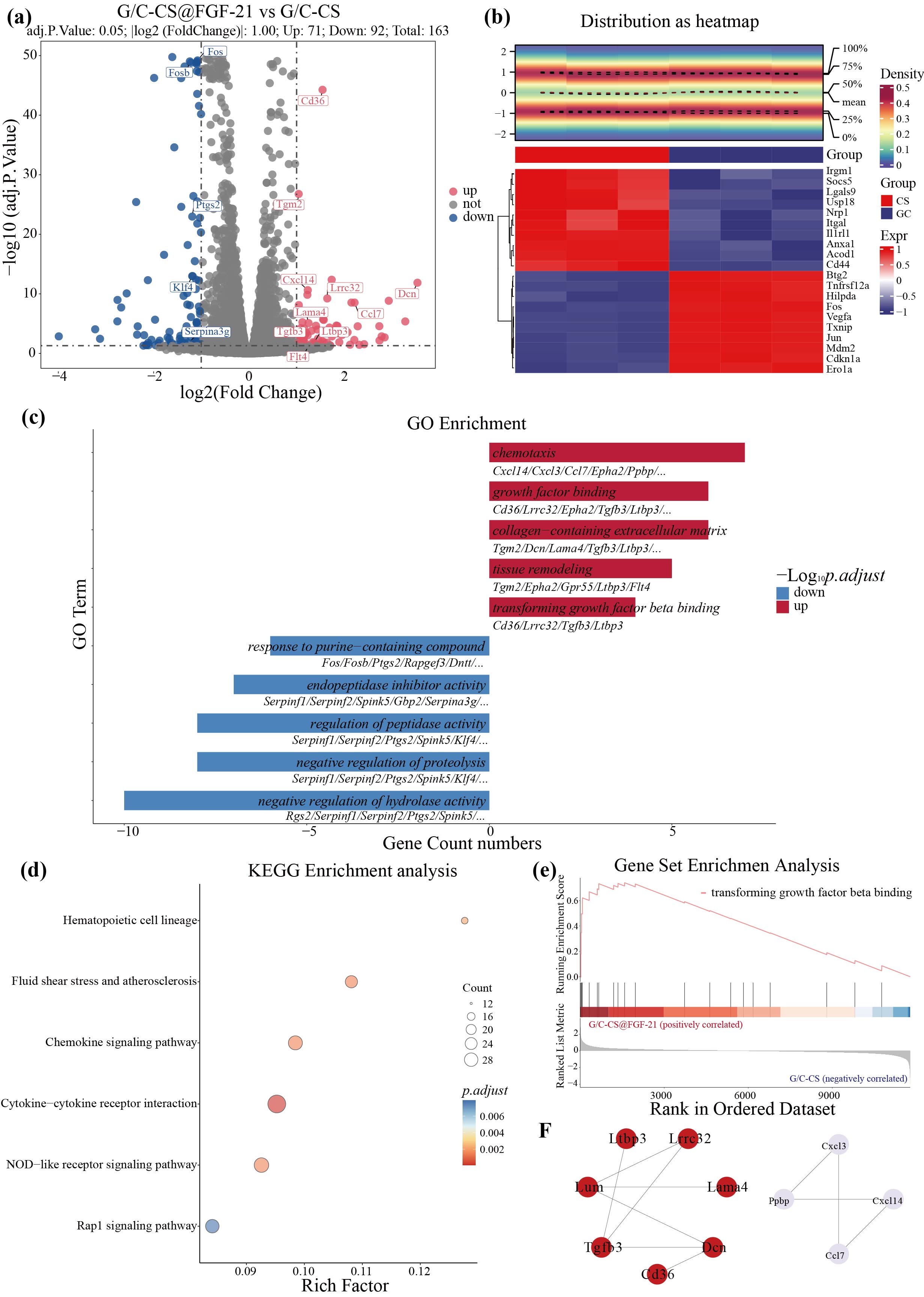


Figure S7. Transcriptional profiles for RAW264.7 cells on G/C-CS and G/C-CS@FGF-21 hydrogels. (a) Volcano plot transcriptional landscape between G/C-CS and G/C-CS@FGF-21. The x-axis represents log2 transformed fold change, and the y-axis shows the log transformed p value adjusted for multiple test correction; (b) The heat map of wound healing and inflammation related genes between G/C-CS and G/C-CS@FGF-21; (c) GO enrichment analysis between G/C-CS and G/C-CS@FGF-21; (d) KEGG enrichment analysis between G/C-CS and G/C-CS@FGF-21; (f) Gene set enrichment analysis between G/C-CS and G/C-CS@FGF-21; (g) Protein-protein interaction networks of differentially expressed proteins between G/C-CS and G/C-CS@FGF-21.


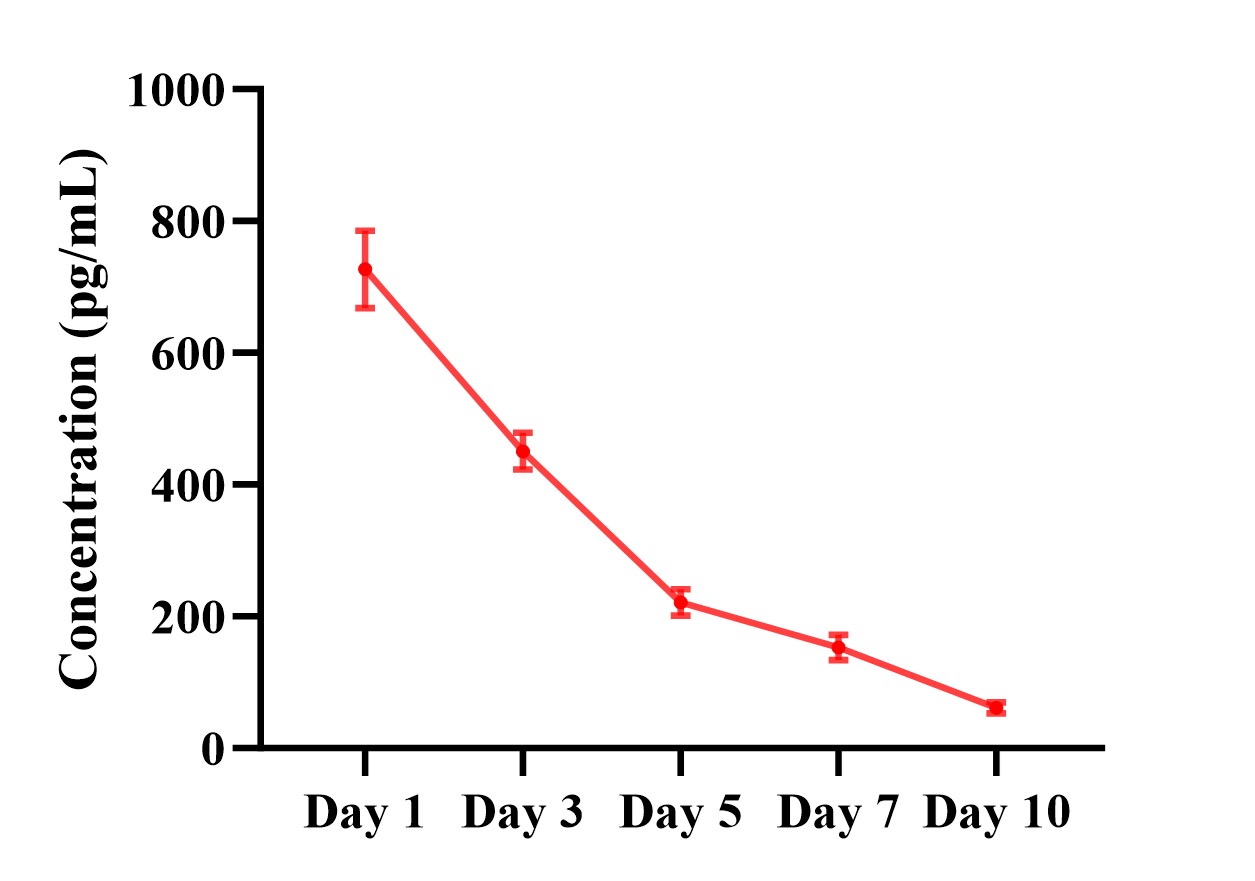


Figure S8. The releasement of FGF-21 from G/C-CS@FGF-21 hydrogel.


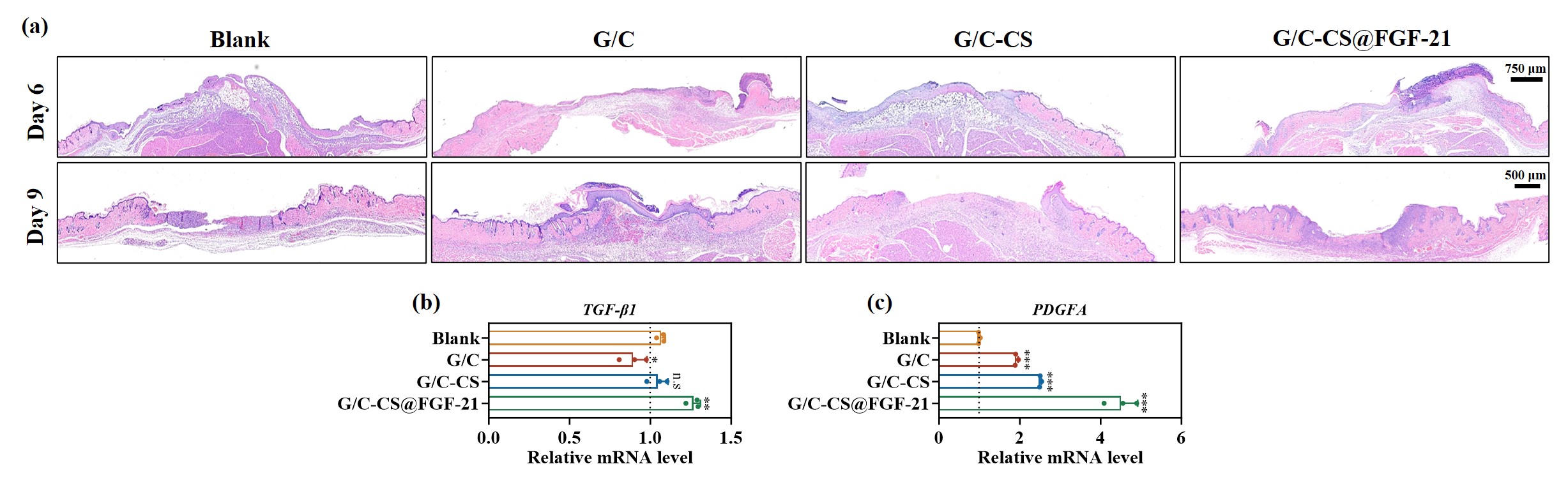


Figure S9. (a) H&E staining of regenerated skin tissues collected on day 6 and 9; (b, c) qRT-PCR results of TGF-β1 and PDGF-α gene expressions for regenerated skin tissues collected on day 15.
